# Life in the Wheat Litter: Effects of Future Climate on Microbiome and Function During the Early Phase of Decomposition

**DOI:** 10.1007/s00248-021-01840-6

**Published:** 2021-09-06

**Authors:** Sara Fareed Mohamed Wahdan, Shakhawat Hossen, Benjawan Tanunchai, Chakriya Sansupa, Martin Schädler, Matthias Noll, Turki M. Dawoud, Yu-Ting Wu, François Buscot, Witoon Purahong

**Affiliations:** 1 Department of Soil Ecology, UFZ-Helmholtz Centre for Environmental Research, Theodor-Lieser-Str. 4, 06120 Halle (Saale), Germany; 2 Botany Department, Faculty of Science, Suez Canal University, Ismailia, 41522 Egypt; 3 Botany and Microbiology Department, College of Science, King Saud University, P.O. Box 2455, Riyadh, 11451 Saudi Arabia; 4 Institute of Ecology and Evolution, Friedrich-Schiller-Universität Jena, Dornburger Str. 159, 07743 Jena, Germany; 5 Department of Applied Sciences, Institute for Bioanalysis, Coburg University of Applied Sciences and Arts, Coburg, Germany; 6 Department of Community Ecology, UFZ-Helmholtz Centre for Environmental Research, Theodor-Lieser-Str. 4, 06120 Halle (Saale), Germany; 7 German Centre for Integrative Biodiversity Research (iDiv) Halle-Jena-Leipzig, Deutscher Platz 5e, 04103 Leipzig, Germany; 8 Department of Forestry, National Pingtung University of Science and Technology, Neipu, Pingtung 91201 Taiwan

**Keywords:** Wheat litter decomposition, Climate change, Litter physicochemical properties, Hydrolytic enzymes, MiSeq Illumina sequencing, Microbial community

## Abstract

Even though it is widely acknowledged that litter decomposition can be impacted by climate change, the functional roles of microbes involved in the decomposition and their answer to climate change are less understood. This study used a field experimental facility settled in Central Germany to analyze the effects of ambient vs. future climate that is expected in 50–80 years on mass loss and physicochemical parameters of wheat litter in agricultural cropland at the early phase of litter decomposition process. Additionally, the effects of climate change were assessed on microbial richness, community compositions, interactions, and their functions (production of extracellular enzymes), as well as litter physicochemical factors shaping their colonization. The initial physicochemical properties of wheat litter did not change between both climate conditions; however, future climate significantly accelerated litter mass loss as compared with ambient one. Using MiSeq Illumina sequencing, we found that future climate significantly increased fungal richness and altered fungal communities over time, while bacterial communities were more resistant in wheat residues. Changes on fungal richness and/or community composition corresponded to different physicochemical factors of litter under ambient (Ca^2+^, and pH) and future (C/N, N, P, K^+^, Ca^2+^, pH, and moisture) climate conditions. Moreover, highly correlative interactions between richness of bacteria and fungi were detected under future climate. Furthermore, the co-occurrence networks patterns among dominant microorganisms inhabiting wheat residues were strongly distinct between future and ambient climates. Activities of microbial β-glucosidase and N-acetylglucosaminidase in wheat litter were increased over time. Such increased enzymatic activities were coupled with a significant positive correlation between microbial (both bacteria and fungi) richness and community compositions with these two enzymatic activities only under future climate. Overall, we provide evidence that future climate significantly impacted the early phase of wheat litter decomposition through direct effects on fungal communities and through indirect effects on microbial interactions as well as corresponding enzyme production.

**Supplementary Information:**

The online version contains supplementary material available at 10.1007/s00248-021-01840-6.

## Introduction

Wheat litter incorporation into the soil is a well-established sustainable practice in modern agricultural systems [1]. Decomposition of wheat residues supports plant productivity by increasing soil organic matter and other nutrients [2]. The dynamics of the plant litter decomposition process is triggered and regulated by several factors such as litter quality, abundances, and activities of soil decomposers, especially saprotrophs. Moreover, all of these factors are governed by climatic conditions [3–6]. Wheat litter consists of cellulose (28–39%), hemicelluloses (23–24%), lignin (16–25%), and few contents of ashes and proteins [7]. The relative proportions of these compounds differ within plant organs and determine litter quality [8]. In cropland ecosystems, it is a common practice that only the glucan- (hemicellulose) and lignin-rich short stems (i.e., 10 cm aboveground) remain after harvest to be incorporated into the soil [7].

Microbes are the primary agents of decomposition, whereby fungi play essential roles due to their ability to produce various enzymes catalyzing breakdowns of polymeric organic plant compounds such as cellulose, hemicellulose, and lignin [9]. Bacteria facilitate decomposition processes directly by producing decomposing proteolytic and cellulolytic enzymes or indirectly by interacting with fungi or by increasing available nutrients for further colonizing taxa [3, 10]. A recent study reported a stronger impact of bacteria on litter decomposition rates than fungi [11]. Numerous studies have demonstrated that the microbial activities, as measured by the amount of microbial enzymes, are linked with litter decomposition rates in different ecosystems [6, 10, 12, 13]. The antagonistic or competitive relationships among litter-colonizing microbes significantly influence the quantities of these litter-degrading enzymes [10, 13]. However, the complex interactions among microbial communities, chemical composition of wheat litter, and enzyme activities remain unclear, especially under the conventional farming practices of cropland ecosystems. In addition, the driving physicochemical factors, identity, and dynamics of microorganisms associated with wheat residues in soil are still poorly documented at the early stage of decomposition. Changes of the microbial community assemblage at the early stage of decomposition determine the microbial richness and community compositions, nutrient cycling, and related ecosystem functions in later stages [14, 15].

At global and regional scales, climatic factors, especially temperature and moisture, are the main driving elements of litter decomposition, as they affect both the identity and activity of decomposing organisms [10, 16, 17]. A large experimental study investigated early stage litter decomposition at the biome scale (across nine biomes) and showed that climatic conditions had a significant role on litter mass loss, whereby precipitation was the more influencing factor over temperature [5]. In agroecosystems, a meta-analysis reported that an increase of temperature by 2 °C accelerated litter decomposition across China [18]. At local scale, high moisture and temperature seasonal variations were found to increase wheat litter decomposition rate [1]. Since litter decomposition is affected by temperature and moisture regimes, future climate conditions including the combined effects of both factors are expected to influence such decomposition process. Only few studies to date have investigated these combined factors on litter decomposition. For instance, Yin et al. reported adverse effect of future climate conditions on litter decomposition rate [19]. To the best of our knowledge, the influence of future climate compared to ambient climate conditions on mass loss of wheat litter and associated microbial communities along with their enzyme activities is poorly investigated.

The present study took advantage of a field infrastructure, the Global Change Experimental Facility (GCEF), established in Germany. This facility has been designed to investigate the consequences of a predicted future climate scenario on ecosystem processes in comparison with ambient climate condition across different land uses [20]. Future climate scenario is based on projections for the next 50–80 years with an increased temperature and a changed precipitation pattern consisting of reduced precipitation in summer and increased precipitation in spring and autumn. We performed a litter decomposition experiment in conventionally managed cropland ecosystem plots. In our preliminary work, we have demonstrated that fungal plant pathogens are highly dominant in wheat residues during the early phase of field decomposition [21]. Pathogenic fungi were accounted by ~ 87% of the relative abundance of total mycobiome, while saprotrophs were present at relatively low abundance (1.1–3.7%) [21]. Some of these plant pathogens are reported to have litter-decomposing abilities [22]. Therefore, the aims of the present study were to compare the influence of ambient and future climatic conditions on (i) the diversity and community composition patterns of both bacteria and fungi inhabiting wheat litter during the early phase of decomposition (2 months after field incorporation); (ii) the litter physicochemical factors related to changes in the bacterial and fungal diversity and community composition; (iii) the interaction between plant pathogenic fungi and other microbes (bacteria and saprotrophic fungi); (iv) the changes of physicochemical properties and mass loss of wheat litter, as well as the activities of microbial enzymes; and (v) the linkage among microbial richness, community compositions, litter physicochemical properties, and enzyme activities. We hypothesized that (H1) patterns of microbial richness and community dynamics would differ under ambient and future climates; (H2) climate changes would lead to a shift in the correlative interactions among bacteria and fungi (total, saprotrophs, and pathogens); (H3) climate change has indirect effect on microbial communities through influencing on wheat litter physicochemical properties; and finally (H4) direct and indirect effects of future climate on microbial communities negatively impact enzyme activities and mass loss of wheat litter during the early phase of decomposition.

## Methods and Materials

### Study Site and Experimental Platform

Our study was carried out in a temperate cropland characterized by a subcontinental climate (mean temperature 8.9 °C, and mean annual rainfall of 498 mm for the period 1896–2013; mean temperature 9.8 °C, and mean annual rainfall of 516 mm for the period 1995–2014) in the Global Change Experimental Facility (GCEF), a field research station of the Helmholtz Centre for Environmental Research in Bad Lauchstädt, Saxony-Anhalt, Germany (51°22′60 N, 11°50′60 E, 118 m a.s.l.). During the study period (2018), the mean temperature was 10.8 °C with an annual rainfall of 254 mm. The GCEF (Fig. S1a) was designed to comparatively investigate the consequences of future climate and ambient climate conditions on ecosystem processes in a 50 field plots (400 m^2^ each), with half of them subjected to ambient and future climatic conditions, respectively [20]. The future climate conditions is a consensus scenario across three models (COSMO-CLM [23], REMO [24], and RCAO [25]) of climate change in Central Germany for the time between 2070 and 2100 that manipulate both precipitation and temperature. In the GCEF, climate manipulation has started in 2014 (4 years prior to our experiment). Future climate plots (Fig. S1b) are equipped with mobile shelters and side panels, as well as an irrigation system; the roofs are controlled by a rain sensor. As result of continuous adjustment of irrigation or roof closing, precipitation is reduced by approximately 20% in summer months and increased by about 10% in spring and autumn. To simulate the increase in temperature with asymmetry between daytime and nighttime warming, we used the standard method passive nighttime warming to maintain the higher daytime temperature for increasing night temperature [26]. The shelters and panels automatically close from sundown to sunrise to increase the mean daily temperature by approximately 0.55 °C. The resulting changes in climate conditions before and during the study period were shown in Figure S2. Ambient climate plots are equipped with the same steel constructions (but without shelters, panels, and irrigation system) to mimic possible microclimatic effects of the experimental setup [20]. The wheat litter decomposition experiment was performed on the conventional farming plots under both ambient (five replicate plots) and future climate (five replicate plots) conditions. The conventional farming plots are characterized by a typical regional crop rotation (including winter rape, winter wheat, and winter barley) with tillage and application of mineral fertilizers and pesticides. Management details are given elsewhere [20].

### Litterbag Preparation, Experimental Design, and Sampling

After harvest of winter wheat (*Triticum aestivum* L.) in 2018, the litter left over (10 cm aboveground) was sampled from each GCEF field plot and placed in sterile plastic bags before transferred to the laboratory on ice. The wheat litter from each plot was oven-dried at 25 °C for 3 days to normalize the moisture content, and then 10 g was enclosed in a litterbag (20 × 15 cm, 5 mm mesh size) [21]. Three litterbags were returned back to each field plot (five ambient conventional farming plots and five future conventional farming plots) in mid-August 2018. To simulate the natural field conditions, the litterbags were placed on the soil surface at the beginning of the experiment and following the agricultural practices (tillage), they were buried at 5 cm depth after 20 days. First sampling occurred at the onset of the experiment (0 days). The second sampling was done in first 30 days and the third sampling at 60 days after field incorporation. For sampling, one litterbag per plot was placed in sterile plastic bag before transferred to the laboratory on ice. Our experiment was therefore established at the end of the drought period under future conditions in summer. Physicochemical properties of soil did not differ significantly between ambient climate and future climate plots (Table S1).

### Microbial DNA Extraction, PCR, and Illumina MiSeq Sequencing

Wheat litter from each bag (a total of 30 bags; 10 bags for each time point where half of these are incubated under ambient and future climate conditions, respectively) was homogenized with the aid of liquid N_2_ and was used for further analyses. DNA was extracted from 150 mg of homogenized wheat litter sample using a DNeasy PowerSoil kit (Qiagen, Valencia, CA, USA) according to the manufacturer’s instructions, then subjected to polymerase chain reaction (PCR). The V5–V7 region of the bacterial 16S rRNA gene was amplified using the following primers: BAC799F forward (5′-AACMGGATTAGATACCCKG-3′) [27] and BAC1193R reverse (5′-ACGTCATCCCCACCTTCC-3′) [28]. These bacterial primer pairs were chosen because they do not amplify the chloroplast DNA in pyrosequencing [29]. The ITS2 region of fungi was amplified using the following primers: fITS7 (5′-GTGARTCATCGAATCTTTG-3′) [30] and ITS4 (5′-TCCTCCGCTTATTGATATGC-3′) [30]. The amplification was performed in a two-step process. First amplifications were performed in 25 µL reactions with a Qiagen HotStar Hi Fidelity Polymerase Kit (Qiagen Inc., Valencia, CA, USA), 1 µL of each 5 µM primer, and 1 µL of DNA template. Reactions were performed on ABI Veriti thermocyclers (Applied Biosystems, Carlsbad, CA, USA). The first PCR conditions were 95 °C for 5 min, then 35 cycles of 94 °C for 15 s, 54 °C for 60 s, 72 °C for 1 min, followed by one extension cycle of 72 °C for 10 min, and 4 °C hold. Amplicons from the first stage amplification were diluted 1:10 and then used as a template in the second PCR. During the second PCR, dual indexes were attached using the Nextera XT Index Kit with the same amplification conditions as the first stage, except for 10 cycles. Amplification products were visualized with eGels (Life Technologies, Grand Island, NY) as explained by the manufacturer. PCR products were then pooled equimolar and each pool was size selected in two rounds using Agencourt AMPure XP (Beckman Coulter, Indianapolis, IN) in a 0.75 ratio for both rounds. Size selected pools were then quantified using the Quibit 2.0 fluorometer (Life Technologies). Sequencing was performed using MiSeq (Illumina, Inc., San Diego, CA) 2 × 300 bp paired-end strategy according to manufacturer’s manual.

### Bioinformatic Analysis

The primer sequences were trimmed from the demultiplexed raw reads using cutadapt [31]. The pair-end raw reads of bacterial and fungal datasets were merged using the simple Bayesian algorithm with a threshold of 0.6 and a minimum overlap of 20 nucleotides as implemented in PANDAseq [32]. Reads fulfilling the following criteria were remained for further analyses: a minimum length of 350 (bacteria) and 120 (fungi) nt; a minimum average quality of 29 (bacteria) or 25 (fungi) Phred score; containing homopolymers with a maximum length of 20 nt; without ambiguous nucleotides. We detected chimeric sequences using the UCHIME algorithm [33] as implemented in MOTHUR and removed from the datasets. The obtained reads were then clustered into operational taxonomic units (OTUs) using the CD-HIT-EST algorithm [34] at a threshold of 97% sequence similarity. The OTU representative sequences (defined as the most abundant sequence in each OTU) were taxonomically assigned against the reference sequences from the SILVA database v132 [35] for prokaryote 16S rRNA gene and the unite database (version unite.v7) [36] using the naive Bayesian classifier as implemented in MOTHUR [37] using the default parameters. Rare OTUs (singletons and doubletons) which potentially might originate from artificial sequences [38] were removed. The read counts were normalized to the smallest read number per sample. Therefore, the final normalized dataset without rare OTUs was used for further statistical analysis, unless otherwise stated. The ecological and metabolic functions of bacterial OTUs were predicted using FAPROTAX [39] and the functional annotation tool of prokaryotic taxa v.1.1, whereas those of fungal OTUs were predicted using FUNGuild [40].

### Mass Loss and Physicochemical Analysis of Wheat Litter

The dry mass of wheat litter samples from the GCEF plots was determined after oven drying at 105 °C to constant weight (mostly after 24 h) and was used for determination of mass loss and moisture content at three time points (0, 30, and 60 days) under ambient and future climate regimes conditions. Approximately 2 g of oven-dried samples (30 samples) was ground and used for detection of litter physicochemical properties. In brief, total C and N concentrations were determined by dry combustion at 1000 °C with a CHNS-Elemental Analyzer (Elementar Analysensysteme GmbH, Hanau, Germany) according to manufacturer’s protocol. Available phosphorus was extracted and measured according to Bray 1 method [41]. Concentration of cations (K^+^, Mg^2+^, Ca^2+^, and Na^+^) was determined by atomic absorption spectrophotometry, using a Z 5300 instrument (Hitachi—Science & Technology, Tokyo, Japan) following recommendations of the manufacturer. The pH of the wheat litter samples was measured using a WTW Multi 3510 IDS portable meter (Weilheim, Germany).

### Assay of Microbial Enzyme Activity in Wheat Litter

Activities of five microbial extracellular enzymes were measured in the same 30 samples of homogenized wheat litter. Of those, three are hydrolytic enzymes important for the acquisition of polymeric carbon (β-glucosidase), nitrogen (N-acetylglucosaminidase), and phosphorus (phosphatase); and two are oxidative enzymes related to the chemical modification of lignin (phenol oxidase and peroxidase) [3]. Hydrolytic and oxidative enzymes were measured based on 4-methylumbelliferone (MUB) derivatives and 3,3′,5,5′-tetramethylbenzidine (TMB), respectively, as described previously [42].

### Statistical Analysis

Statistical analyses were performed using the PAST program v.2.17c [43] and IBM SPSS Statistics (version 24) software. All the analyses were conducted based on five independent replicate plots of the field experiment (*n* = 5) with time as within plot factor. Bacterial and fungal OTU richness were calculated for each sample using the “diversity” function in the PAST program. Sample rarefaction curves of fungi and bacteria are shown in Fig. S3. As the rarefaction curves indicated sufficient OTU coverage, we used the observed OTU richness directly as a proxy for bacterial and fungal diversity. Permutational multivariate analysis of variance (NPMANOVA) [44] based on Jaccard distance (permutations = 999) was performed to test the impact of sampling times and climate conditions on bacterial and fungal (including plant pathogen and saprotrophs) communities over time. Non-metric multidimensional scaling (NMDS) was used to visualize the variations of bacterial and fungal community compositions among the three sampling time points (0, 30, and 60 days), under ambient and future climate conditions, respectively. We used the presence/absence data of bacterial and fungal communities and Jaccard dissimilarity distances (permutations = 999) to perform the NMDS ordination plot as they are more reliable than relative abundance data. All physicochemical properties that significantly affected bacterial and fungal community compositions (*p* < 0.05) were fitted in the respective NMDS ordination plots using PAST. *T*-test was applied to evaluate the effect of climate on mass loss of wheat litter at 30 and 60 days under ambient and future climate conditions, respectively. Effects of climate, time, and their interaction on physicochemical properties of wheat litter and on microbial enzyme activity were assessed by time-series analysis using SPSS as the data sets vary over time. With this test, climate was used as a between‐subject factor and time was used as within plot factor. We tested the correlation between bacteria and fungi (community composition and richness) and wheat litter physicochemical properties. Additionally, the correlation between microbial communities and richness and enzyme activities was investigated. The first and second axis scores of NMDS were used to represent the bacterial or fungal community composition. For correlation analyses, Jarque–Bera test was performed to evaluate the distribution of the datasets [45]. Pearson’s correlation and Spearman’s rank correlation were applied with normally distributed and non-normally distributed datasets, respectively. Due to the significance of fungal plant pathogens in field-incorporated wheat litter, we aimed to characterize the interactions between these pathogens and other microorganisms colonizing wheat residues. We performed ecological network analysis (ENA) using Spearman’s rank correlations (*p* < 0.05) for ambient and future climate conditions, separately. The ecological networks of potential interacting taxa were visualized using cytoscape 3.0.2. [46]. Network properties were calculated using network analyzer as implemented in cytoscape. These correlation networks included nodes that consisted of plant pathogenic fungi and fungal and bacterial OTUs as a proxy for “species,” while the edges represented the correlative relations among the OTUs [47]. Microbial hubs are defined as strongly interconnected taxa, which can have a severe effect on microbial community compositions and networks if they were removed [48].

## Results

### General overview of datasets

A total of 237,675 quality-filtered bacterial 16S rRNA gene and 585,217 fungal ITS sequence reads were obtained, resulting in rarefied 5104 bacterial and 9020 fungal sequence reads per sample (Fig. S3). Bacterial communities under both ambient and future climate conditions were dominated by Proteobacteria (85–86% of the total bacterial sequences reads), which were mostly assigned to Gammaproteobacteria (95–96%) and Alphaproteobacteria (4–5%), and Actinobacteria (11–13% of the total bacterial sequences, with 89–92% assigned to Micrococcales). Fungal communities were dominated by Ascomycota (98.3% of total fungal sequences reads), while Basidiomycota was rare (1.7% of all fungal sequences) at the early phase of wheat litter decomposition. Information on bacterial and fungal taxonomic compositions are provided in Figs. S4 and S5. The datasets comprised of 2737 bacterial and 275 fungal OTUs. 15.9% and 28.7% of bacterial and fungal OTUs, respectively, were found only under the future climate condition.

#### Impact of Future Climate Condition, Time, and Their Interaction on Microbial Richness in Wheat Litter

We investigated the impact of climate, field incorporation time, and their interaction on bacterial and fungal OTU richness (Fig. S6). Future climate condition significantly increased the richness of fungal OTUs (*F* = 16.32, *p* < 0.001) in wheat litter. Both bacterial (*F* = 27.80, *p* < 0.001) and fungal (*F* = 13.60, *p* < 0.001) OTU richness increased over time.

#### Microbial Community Composition in Wheat Litter Over Time in Correspondence to Future Climate Condition

**Fig. 1:**
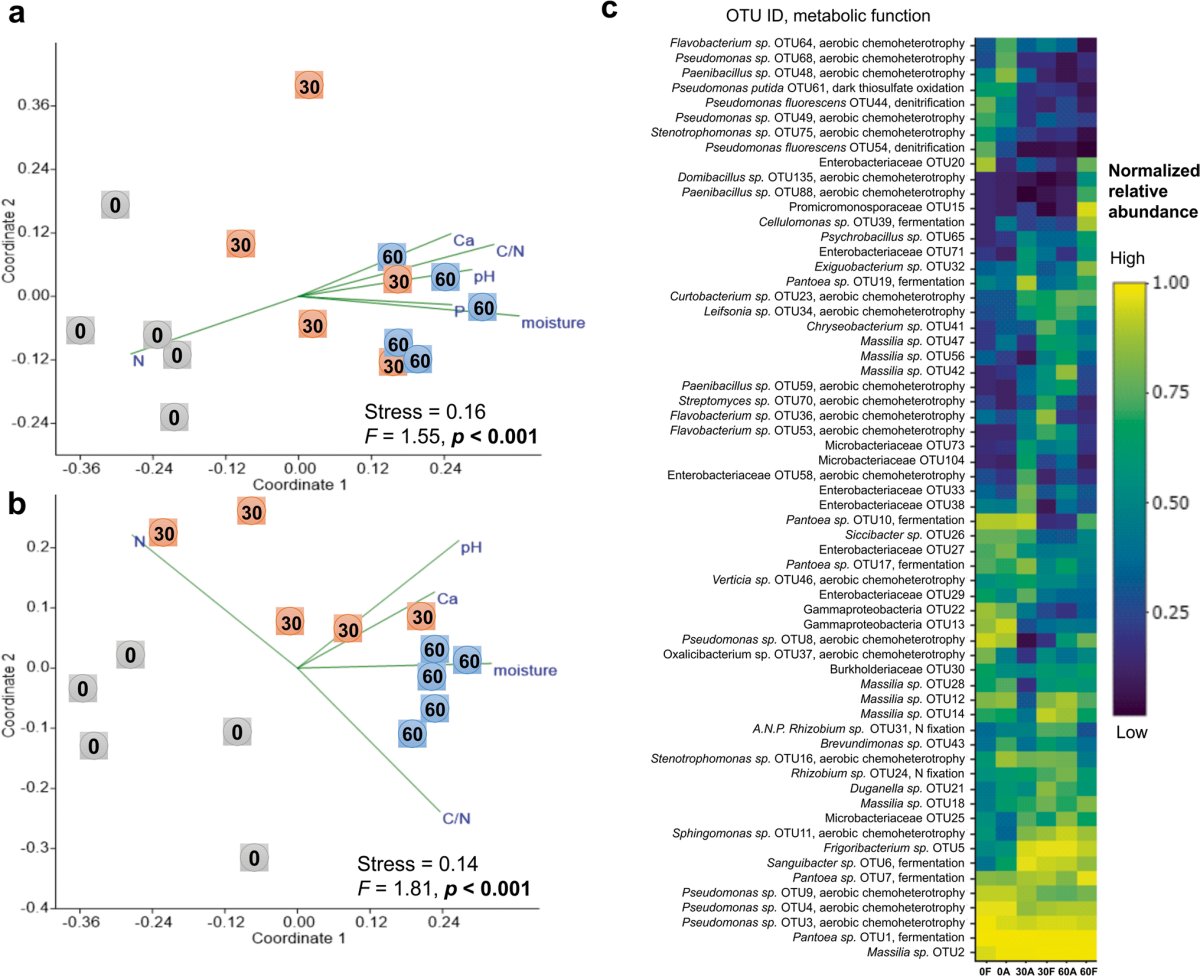
Bacterial community composition in wheat litter at the early stage of decomposition. Non-metric multidimensional scaling (NMDS) ordination diagrams of the bacterial community colonizing wheat litter residues under (**a**) ambient and (**b**) future climate conditions over time. NMDS ordination based on Jaccard dissimilarities was used to determine the compositional variation of all bacterial OTUs detected at different sampling times. In NMDS ordinations, the numbers 0, 30, and 60 represent the sampling time in days. Significant effect of time (*p* < 0.05) based on NPMANOVA is indicated in bold. All community-shaping wheat litter physicochemical properties (*p* < 0.05) were plotted in the respective NMDS ordination plots. (**c**) Normalized heat map of 62 dominant bacterial OTUs, which account for at least 1% of the relative abundance at one or more sampling times. A, ambient climate; F, future climate

**Fig. 2:**
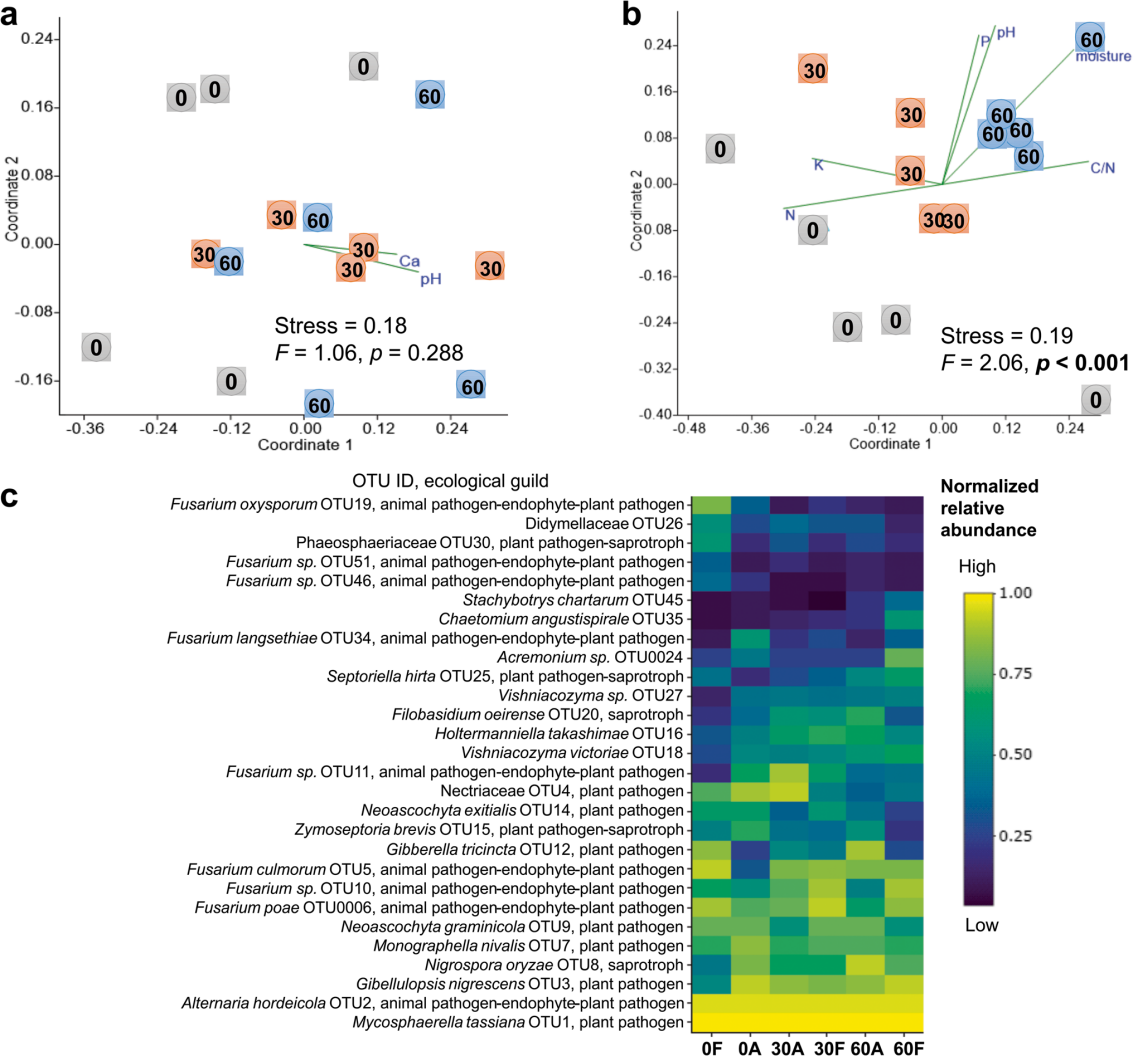
Fungal community composition in wheat litter at the early stage of decomposition. Non-metric multidimensional scaling (NMDS) ordination diagrams of the bacterial community colonizing wheat litter residues under (**a**) ambient and (**b**) future climate condition over time. NMDS ordination based on Jaccard dissimilarities was used to determine the compositional variation of all bacterial OTUs detected at different sampling times. In NMDS ordinations, the numbers 0, 30, and 60 represent the sampling time in days. Significant effect of time (*p* < 0.05) based on NPMANOVA is indicated in bold. All community-shaping wheat litter physicochemical properties (*p* < 0.05) were plotted in the respective NMDS ordination plots. (**c**) Normalized heat map of 28 dominant fungal OTUs, which account for at least 1% of the relative abundance at one or more sampling times. A, ambient climate; F, future climate

The influences of climate condition, field incorporation time, and their interactions on the microbial community composition colonizing wheat litter were examined at the OTU level (97% identity) Figs. 1 & 2, (Table S2). NPMANOVA based on Jaccard distance revealed that future climate significantly (*F* = 1.45, *p* = 0.015) altered only the fungal community composition and the dynamics of fungal colonizers over time (*F* = 2.06, *p* <0.001) (Fig. 2a, b; Table S2). We recognized a distinct fungal community pattern in each single sampling time only under future climate condition (Fig. 2b). On the other hand, bacterial community composition was shaped only by field incorporation time (*F* = 2.25, *p* = 0.001; Table S2). The most abundant (> 1% of the relative abundance in at least one of the sampling times) microbial taxa present in the early phase of litter decomposition belonged to diverse taxonomic groups with a shift in their relative abundance among sampling times (bacteria, Fig. 1c; fungi, Fig. 2c). However, some taxa were highly abundant at all time points under both climatic conditions such as *Pantoea* (bacterial OTU1), *Massilia* (bacterial OTU2), *Mycosphaerella tassiana* (fungal OTU1), and *Alternaria hordeicola* (fungal OTU2). The FAPROTAX predicted specific metabolic functions (N-fixation, denitrification, fermentation, and thiosulfate oxidation) for the bacterial colonizers (Fig. 1c), while FUNGuild annotation revealed that the fungal community was dominated by potential plant pathogens (Fig. 2c).

#### Co-occurrence Networks Between Plant Pathogenic Fungi and Other Microorganisms Are Dissimilar Between Ambient and Future Climate Conditions

**Fig. 3:**
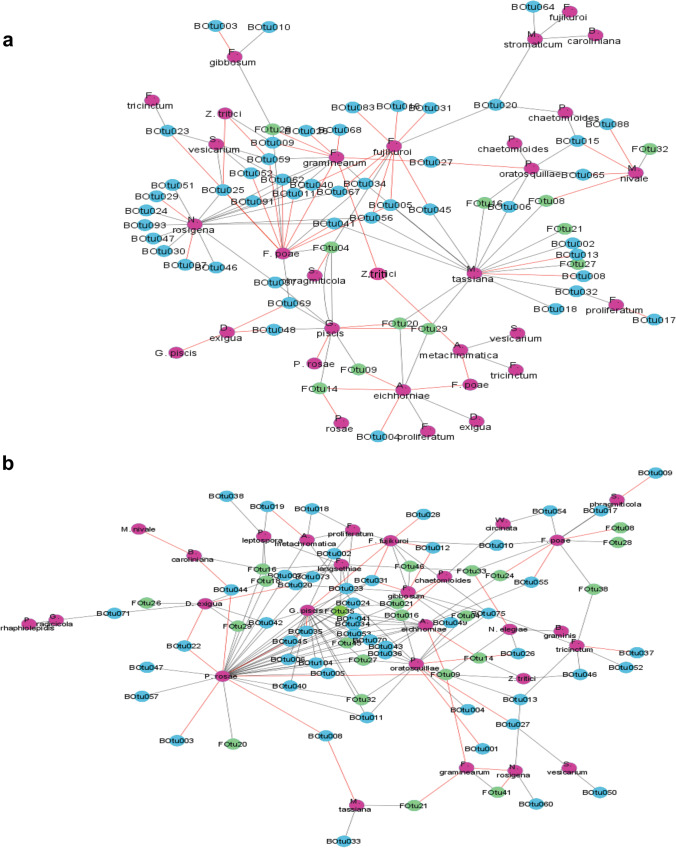
Ecological interaction networks of plant pathogenic fungi and other microorganisms associated with wheat litter at the early stage of decomposition under ambient (**a**) and future climate (**b**) conditions. Plant pathogenic fungal, dominant bacterial, and dominant fungal OTUs are indicated by pink, blue, and green colors, respectively. Color of edges represents co-presence (black) and mutual exclusion (red) correlations

First, we performed a correlation analysis to explore the influence of climate changes on the co-occurrence of bacteria and total fungi as well as for the dominant fungal guilds (saprotrophic and plant pathogenic fungi) (Table 1). Bacterial richness significantly correlated with total and saprotrophic fungal richness under both climate conditions; however, the correlation under future climate condition was clearly stronger (*R* = 0.91–0.92, *p* < 0.001) as compared to the ambient one (*R* = 0.54–0.57, *p* < 0.05). Moreover, bacterial richness correlated with plant pathogenic fungi only under future climate (*R* = 0.55, *p* < 0.05). Due to the economic significance of plant pathogenic fungi colonizing field-incorporated wheat litter, we performed ecological network analyses (ENA) to predict the potential interactions among the dominant plant pathogenic fungi (~ 87% of total fungal sequence read abundance) and further members of the microbial communities under each climate condition, separately (Fig. 3). The network of ambient climate condition consisted of 91 microbes (nodes) and 129 correlations (edges), and the highest number of connections was detected between the fungal pathogens, *M. tassiana* and *Neosetophoma rosigena*, with other microbes (Fig. 3a). The network of future climate condition was consisted of 100 nodes and 170 correlations and the highest number of connections was between the fungal pathogens *Pseudopithomyces rosae* and *Gibellulopsis piscis* with other microbes (Fig. 3b, Table S3 & S4).

**Table 1:** Correlations between bacterial and fungal (total, plant pathogenic, and saprotrophic) richness under each climate condition, separately. Significant correlations are indicated in bold (*p < 0.05, **p < 0.01, ***p < 0.001)

Richness correlation patterns	Ambient	Future
Total bacteria-total fungi	*0.54	***0.91
Total bacteria-plant pathogenic fungi	0.18	*0.55
Total bacteria-saprotrophic fungi	*0.57	***0.92
Total fungi-plant pathogenic Fungi	**0.63	**0.69

#### Effect of Climate Change and Time on Mass Loss, Physicochemical Properties, and Microbial Enzyme Activities of Wheat Litter

**Fig. 4:**
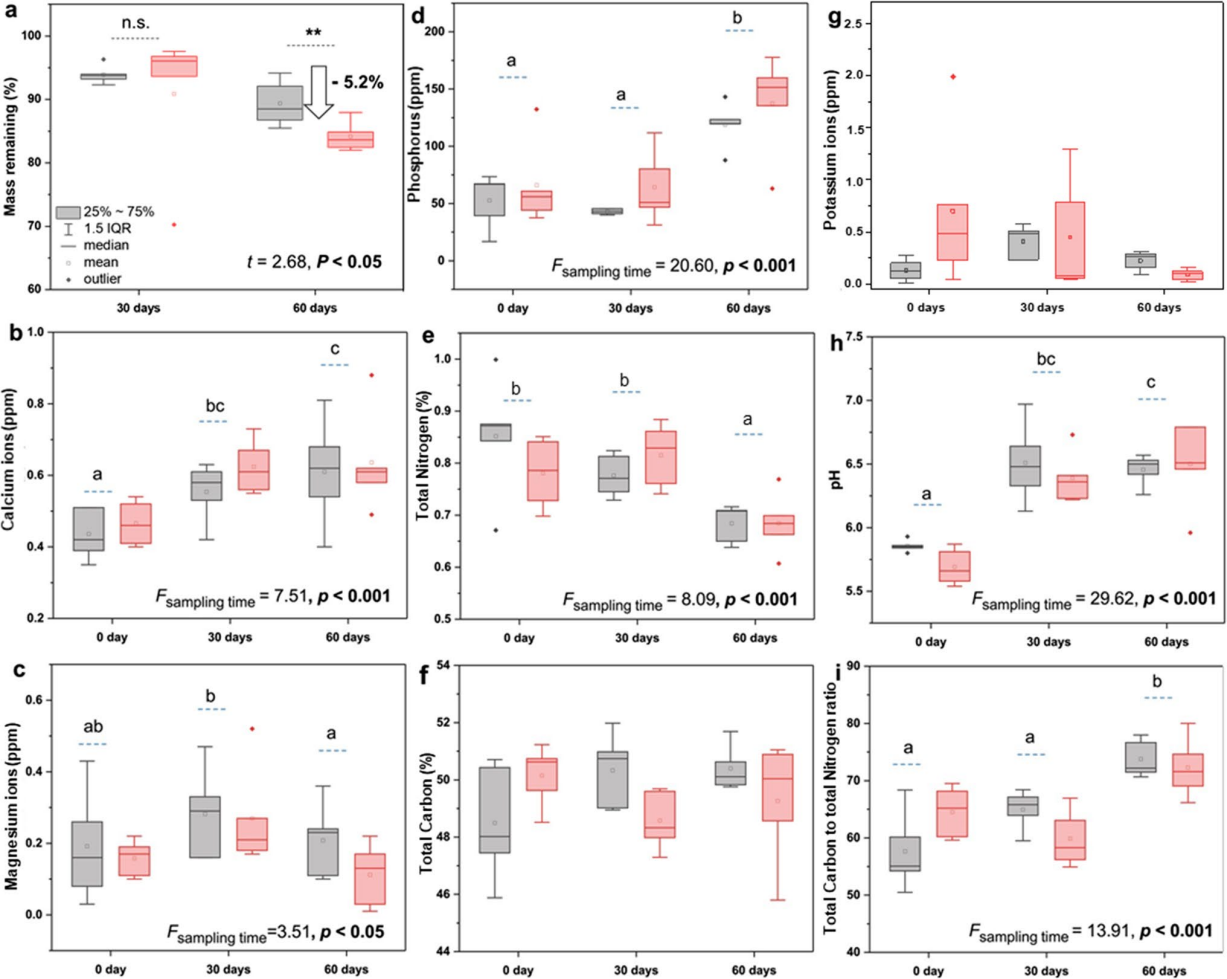
Effects of climate conditions and time on (a) wheat litter dry mass loss, (b–i) physicochemical properties of litter during the early phase of decomposition process. Different letters indicate significant differences following Tukey’s HSD at *p* < 0.05 with separate comparisons indicated by lowercase letters. n.s. represents non-significance, and asterisks represent significant differences (*p* < 0.05) between the two climate treatments within each sampling time (grey bar = ambient climate condition; red bar = future climate condition)

Future climate condition significantly accelerated the mass loss of wheat litter at the early phase of decomposition. After 60 days of field incorporation, wheat litter dry matter was reduced by 15.8% under future climate, compared to 10.6% under the ambient one (*t* = 2.68; *p* < 0.05) (Fig. 4a). With regard to the physicochemical properties of litter (Fig. 4b–i), slow nutrient release was observed during the early phase of decomposition, except for N (*F* = 8.09, *p* < 0.001) and Mg^2+^ (*F* = 3.51, *p* < 0.05) that showed a significant reduction after 60 days of field incorporation. Immobilization of P and Ca^2+^ was clearly observed as the concentrations of both elements increased over time. Moreover future climate, these conditions had a negligible influence on changes of chemical composition of litter. Although we detected a higher release of C and K^+^ at 30-day sampling under future climate (during summer drought), a bioaccumulation of both elements was observed at 60 days (increased precipitation in autumn) to resemble those under ambient climate condition.

**Fig. 5:**
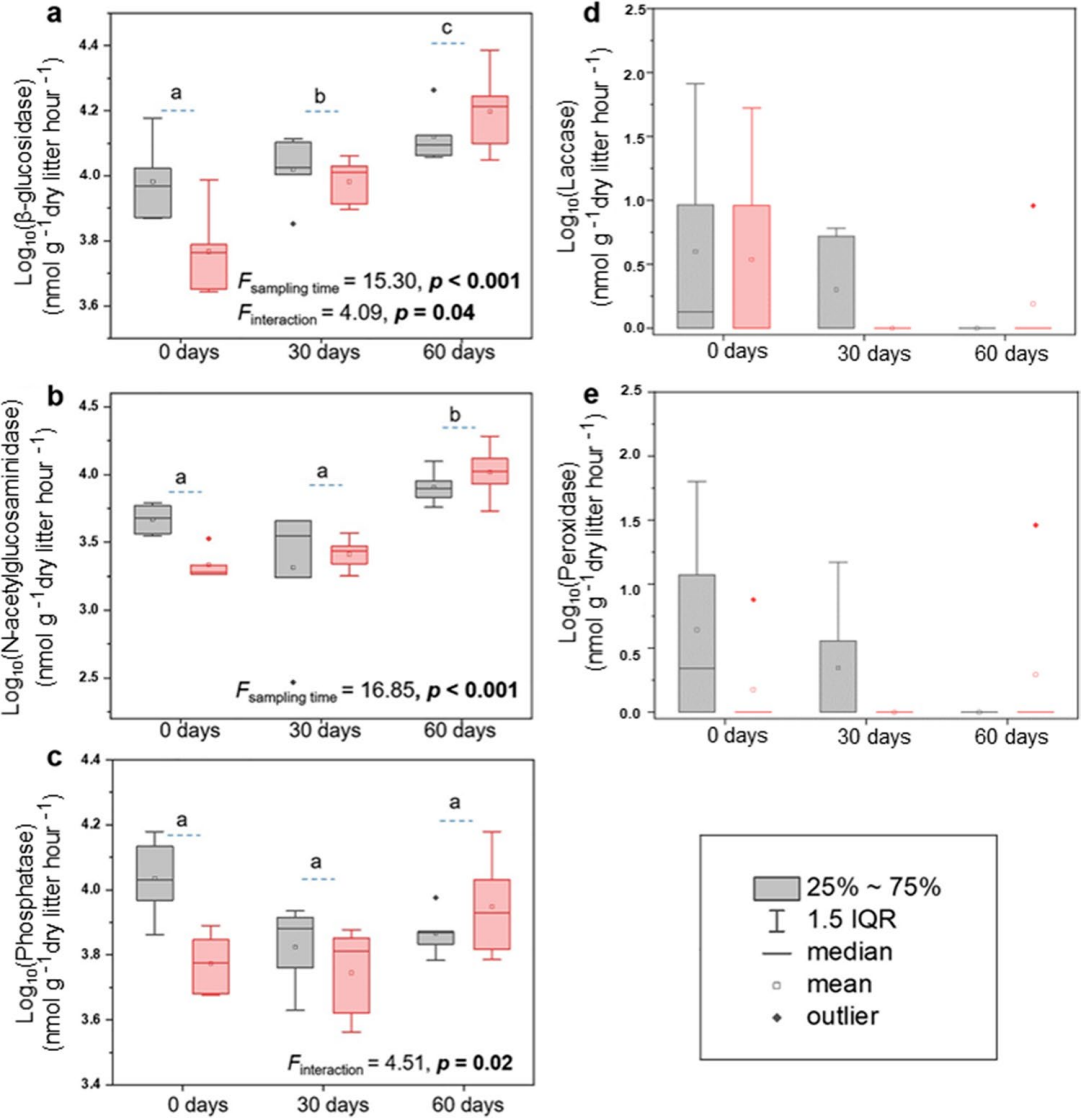
Effects of climate conditions and time on (a–c) hydrolytic and oxidative (d, e) enzyme activities in wheat litter during the early phase of decomposition. Different letters indicate significant differences following Tukey’s HSD at *p* < 0.05. Significant results (*p* < 0.05) are indicated in bold (grey bar = ambient climate and red bar = future climate). Box plot details are also denoted

Analysis of microbial extracellular hydrolytic enzyme activities in wheat litter revealed a significant increase of β-glucosidase (*F* = 15.30, *p* < 0.001) and N-acetylglucosaminidase (*F* = 16.85, *p* < 0.001) over time (Fig. 5a, b). Both enzymes reached the highest activity at 60-day sampling under future climate condition. Almost no activity of oxidative enzymes was detected during the early phase of litter decomposition under both climate conditions (Fig. 5d, e).

#### Influence of Future Climate Condition on the Relationship Between Resident Microbes (Richness and Community Composition) and Physicochemical Properties as well as Microbial Extracellular Enzymes in Wheat Litter

A correlation analysis was performed to elucidate the potential link between wheat litter physicochemical properties and community compositions and richness of litter-resident microbes under both climate conditions (Table 2). The contribution of future climate to influence the factors shaping microbial communities in wheat litter was highly recognized for fungi. For instance, total fungal community composition was significantly (*p* < 0.05) correlated with Ca^2+^, and pH under ambient climate condition, while it was also correlated with C/N, N, P, K^+^, pH, and litter moisture content under future climate condition (Table 2 and Fig. 2a, b). Total fungal richness was significantly correlated with C/N, N, Ca^2^+, pH and moisture under future climate, whereas none of the measured factors correlated significantly with total fungal richness under ambient climate. The same observation was also found in case of saprotrophic and plant pathogenic fungi (Fig. S7). For bacteria, there were a significant correlation between the richness and community composition and common (Ca^2+^, C/N, N, pH, moisture), but also differentiated factors (P) in correspondence to climate condition (Table 2 and Fig. 1a, b).

**Table 2:**
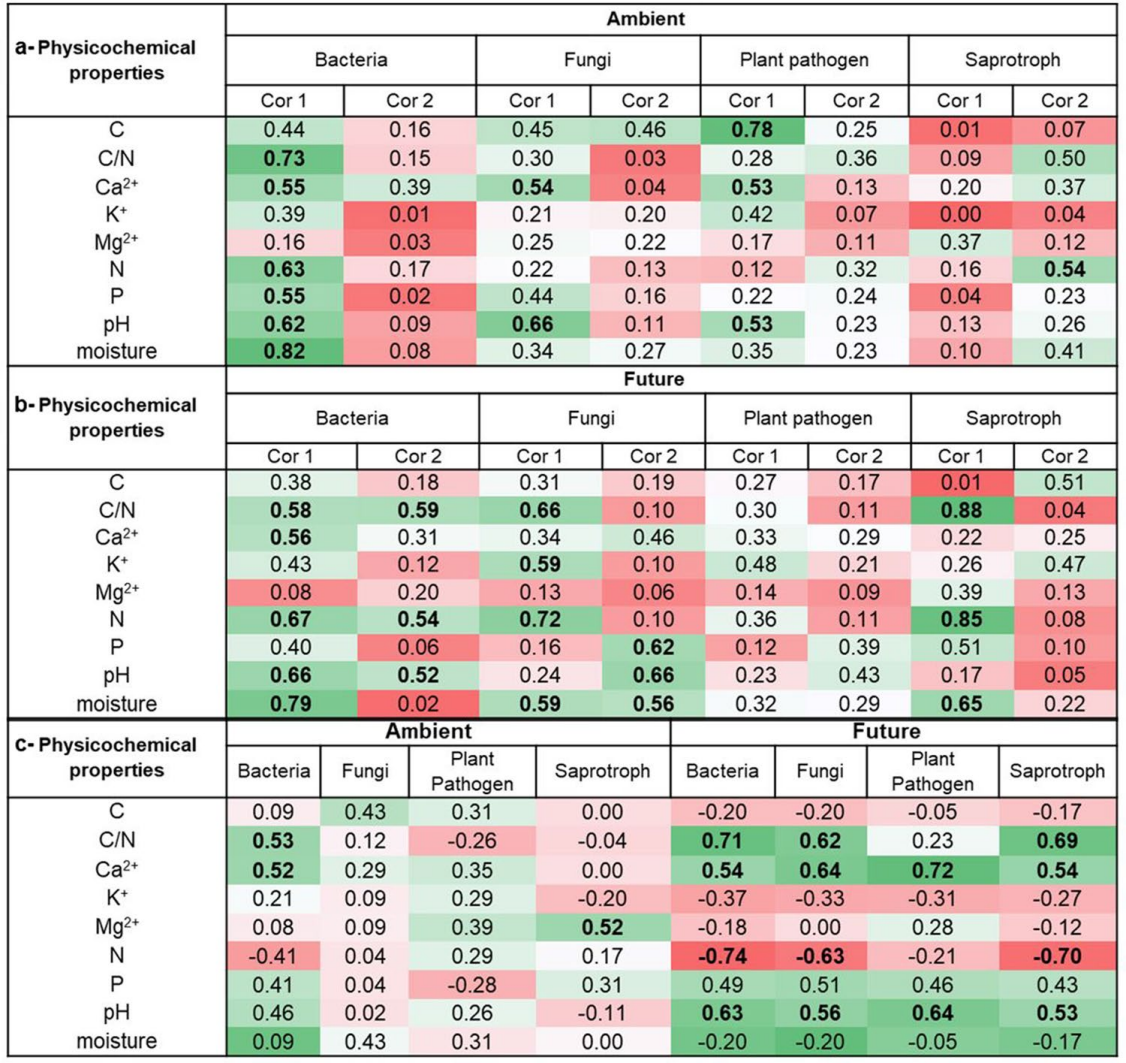
Heat table showing the correlation coefficient values between microbial communities (coordinate1 and coordinate2 of NMDS) and physicochemical properties under (a) ambient and (b) future climate conditions and between (c) microbial richness and physicochemical properties. Cor represents the coordinate. Significant values (*p* < 0.05) are indicated in bold. Bacteria = total bacteria, Fungi = total fungi, Plant pathogen = plant pathogenic fungi, Saprotroph = saprotrophic fungi. Green and red colours indicate values above and below 50th percentile, respectively

A correlation analysis was performed between microbial communities and enzyme activities under both climate conditions (Table 3). It could be considered a potential prediction of ecosystem functions under ambient and future climate conditions. Climatic conditions significantly influenced the correlation between both bacterial and fungal community compositions and enzyme activities in wheat residues. For instance, the bacterial and total fungal community compositions and richness were significantly correlated (*p* < 0.05) with phosphatase activity under ambient climate condition, while they correlated with β-glucosidase and N-acetylglucosaminidase activities under future climate condition. Similarly, saprotrophic and plant pathogenic fungi were found to positively correlate with β-glucosidase and/or N-acetylglucosaminidase only under future climate.

**Table 3:**
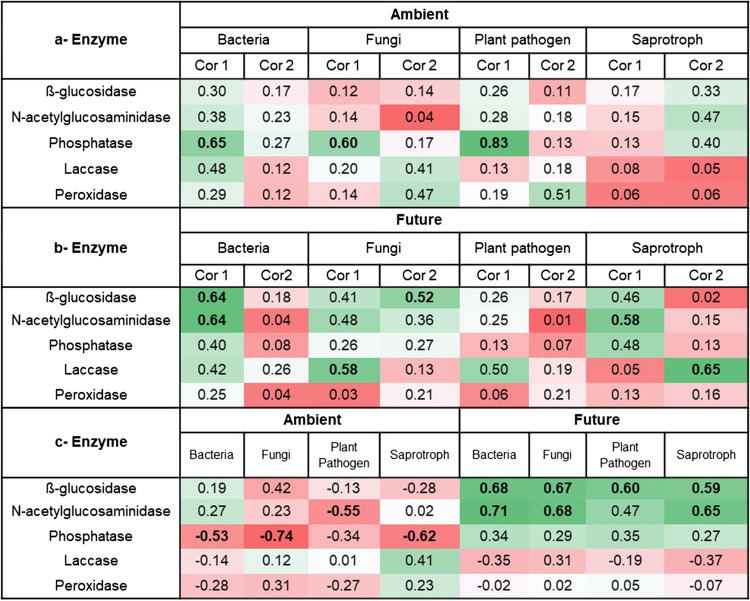
Heat table showing the correlation coefficient values between microbial communities (coordinate1 and coordinate2 of NMDS) and enzyme under (a) ambient and (b) future climate conditions and between (c) microbial richness and enzyme. Cor represents the coordinate. Significant values (*p* < 0.05) are indicated in bold. Bacteria = total bacteria, Fungi = total fungi, Plant pathogen = plant pathogenic fungi, Saprotroph = saprotrophic fungi. Green and red colours indicate values above and below 50th percentile, respectively.

## Discussions

### Differential Responses of Wheat Litter Fungi and Bacteria to Future Climate Condition

Our work highlights major changes in the bacterial and fungal richness and community compositions due to projected climate changes at the early phase of wheat litter decomposition. Since microbes differ in physiology, growth rates, and evolutionary adaptations, they respond differentially in the face of environmental stress. Here, we found that future climate significantly altered fungal community composition and shaped its patterns over time with an increasing species richness. Moreover, future climate condition significantly altered the correlation between fungi and wheat litter physicochemical properties (hypotheses 1). Our results proved the sensitivity and obvious turnover in the community composition of litter-inhabiting fungi due to climate changes. Climate manipulation at the GCEF resulted in altering of precipitation patterns as well as an average increase in daily mean soil temperature by 0.55 °C in the future climate plots. Soil warming is directly impacting microbial metabolism, respiration, and growth, and the processes microbes mediate which are temperature sensitive [49, 50]. Moreover, drought stress forces microbes to shift energy, respiration, and resources allocation from growth to survival mode. For instance, they may accumulate balancing osmolytes to reduce the water potential and maintain hydration. Such mechanism might lead to a shift in the soil community composition because the physiological costs associated with drought stresses depend on microbial inherent resistance and acclimation abilities which is genus species specific [51]. It was noteworthy that our results are in line with the recent global investigation of fungal distribution in soils across biomes, which revealed that temperature and precipitation are the primary and secondary elements of climate variables explaining fungal variation, respectively [52]. On the other hand, future climate did not significantly influence bacterial richness or community composition in wheat residues. However, a positive correlation between bacterial richness and extracellular enzyme activities only under future climate was found (Table 3), which is indicative for a genomic and post-transcriptomic imprint as part of the climate response. Likewise, a comparative study tested the influence of climate change on bacterial communities colonizing plant litter, and drought was found to reduce bacterial biomass; however, metagenomics sequences revealed an increase of different glycoside hydrolase gene families that encode enzymes breakdown various polymers such as cellulose and chitin [53].

Although, future climate conditions had no significant direct effect on initial wheat litter physicochemical factors (hypothesis 3), the correlation between fungi and wheat litter physicochemical factors altered. Under future climate, fungi correlated with C/N, N, P, K^+^, Ca^2+^, and pH. Elements such as N, Ca, and P have fast mineralization rate in litter [6]. Moreover, both Ca^2+^ and pH help in stabilization of organic matter [54].

The impact of future climate on microbial interactions is so far less studied. In the present study, we detected stronger correlative interactions between bacterial and fungal OTU richness under future climate condition (hypotheses 2), which support our interpretation that these interactions help the communities to stand against environmental stress. The result of co-occurrence networks in our study highlighted the influence of climate change on the interaction patterns between bacteria, saprotrophic fungi, and pathogenic fungi. Each network could be defined as “pathobiome”: the plant pathogens and associated microbes that may influence its persistence, transmission, and evolution [55]. The microbial networks showed that correlations (positive or negative) between pathogenic fungi and other soil microbes have been altered in correspondence to climate conditions. Under ambient climate, the highest number of connections was detected between the fungal pathogens, *M. tassiana* and *N. rosigena*, with other microbes, while the highest number of connections was between the fungal pathogens *P. rosae* and *G. piscis* with other microbes under future climate. Our results suggest that global warming might enhance the pathogenic pressure on main crop species, which is a potential threat for future agriculture. Disease expression seems to be the result of an imbalance between a potentially pathogenic species and the rest of the microbial communities, rather than just the presence of the pathogenic species [55].

### Future Climate Positively Affects Mass Loss of Wheat Residues at the Early Phase of Decomposition

We expected that the reduction of precipitation during the summer period coupled with higher temperature of future climate might cause a significant reduction of litter decomposition. However, in contrast to our fourth hypothesis, we found faster mass loss of wheat litter under future (~ 15%) climate as compared to the ambient one (~ 10%). The reason could be that we started our experiment on mid-summer period, which is characterized by increased temperature and reduced precipitation rate at the future climate plots in the GCEF [20]. The mass loss was measured during autumn, which is characterized by higher soil temperature and increase in precipitation by 10% as compared to ambient climate plots. Therefore, we covered the period of increased precipitation together with increasing soil temperature, which coincidences with a faster decomposition rate. Thus, the decomposition rate in our study is indicative only for a certain period of the year when future climate might increase decomposition. Therefore, we hypothesize that the positive effect of future climate on accelerating wheat litter mass loss during the early stage decomposition in the present study, may become inversed at later stages of litter decomposition. On other hand, we found that the mass loss values of wheat stem were only between 10 and 15% after 60 days incubation, which is much lower than the mass loss as reported in other studies [56]. For instance, wheat litter mass loss reported to be 80% after incorporation in rice–wheat rotation system. This result was explained by the high soil moisture content and temperature during the rice cultivation season. Therefore, climate conditions are considered to be the predominant factor when litter quality is similar. Our results suggest that litter decay, an important ecosystem function, would change dramatically under future climate. This will lead to changes in soil C stocks; in turn, altering of soil organic carbon stock will have a major impact on future atmospheric CO_2_, C sequestration, and climate.

### Climate Drives the Contribution of Microbes to the Litter Decomposition Process

Beside other soil biota, microorganisms are considered the engines of plant litter decomposition processes. Future climate condition positively affected mass loss of wheat litter at the early stage of decomposition directly by altering fungal community composition and richness as reported before [11]. In addition, we also detected other effects of climate on microbial activities that contributed to the decomposition process. At the early phase of litter decomposition, labile compounds like simple sugars and amino acids are normally consumed first by microbes with the aid of hydrolytic enzyme activities [3]. This corresponds to the strong positive correlation between microbial communities (both fungi and bacteria) to β-glucosidase and N-acetylglucosaminidase enzyme activity we found only under future climate condition. Similarly, previous study has reported that increasing temperature altered decomposition rates by shifting microbial extracellular enzyme activities [57]. We also showed that a more complex compound such as lignin may not sufficiently decompose at the early phase as lignin degrading fungi were rarely detected and oxidative enzyme activities were negligible in all samples. Though future climate condition had no significant direct effect on initial wheat litter physicochemical factors (hypothesis 3), its interaction with sampling time significantly affected enzyme activities. Decomposition process of plant materials is known to be regulated by fungal-fungal interactions [9] as well as by cross-kingdom relationships between fungi and bacteria [3, 58]. Therefore, changing of plant pathogenic fungal-bacterial and plant pathogenic fungal-fungal and pathogen-pathogen interactions may also be responsible for the different proportions of wheat litter mass loss under future and ambient climate conditions. The majority of plant pathogenic fungi that highly dominate in the early decomposition stage of wheat residue (~ 87% of total detected sequences) in this study have been reported to live as decomposers and some of them are even listed as efficient cellulose decomposers [59]. In a word, our study reported that future climate shifts the fungal community composition and richness over time leading to altering interactions among microbial taxa and hence altered the corresponding function (litter decomposition) of the ecosystem.

## Conclusions

This study provides the first comprehensive view of microbiome (including both bacteria and fungi) associated with the early stage of wheat residue decomposition in ambient and future climate conditions using high-throughput DNA sequencing technique. The strategy developed here can be viewed as a proof-of-concept focusing on wheat residues as a particularly microbial rich ecological compartment, with various patterns of diversity-structure-ecosystem functioning of fungal and bacterial taxa colonizing wheat litter under ambient and future climate treatments. Fungal communities associated with the early phase of wheat residue response much stronger to the future climate change than bacterial communities. Our findings pave the way for a deeper understanding of the complex interactions among pathogens, and other microbial communities as well as effect of climate change on decomposition rate via the microbes and their associated ecosystem functions.

## Supplementary Information

Below is the link to the electronic supplementary material.

Supplementary file1 (DOCX 4456 KB)

Supplementary file2 (XLSX 22 KB)

Supplementary file3 (XLSX 14 KB)

## Data Availability

The bacterial 16S and fungal ITS2 raw read sequence datasets were deposited in the National Center for Biotechnology Information (NCBI) Sequence Read Archive (SRA) under bioproject number PRJNA700837.

## References

[1] Jin Z, Shah T, Zhang L, Liu H, Peng S, Nie L (2020) Effect of straw returning on soil organic carbon in rice–wheat rotation system: a review. Food Energy Secur 9. 10.1002/fes3.200

[2] Zhao X, Yuan G, Wang H, Lu D, Chen X, Zhou J (2019). Effects of full straw incorporation on soil fertility and crop yield in rice-wheat rotation for silty clay loamy cropland. Agronomy.

[3] Purahong W, Wubet T, Lentendu G, Schloter M, Pecyna MJ, Kapturska D, Hofrichter M, Kruger D, Buscot F (2016). Life in leaf litter: novel insights into community dynamics of bacteria and fungi during litter decomposition. Mol Ecol.

[4] Cornwell WK, Cornelissen JH, Amatangelo K, Dorrepaal E, Eviner VT, Godoy O, Hobbie SE, Hoorens B, Kurokawa H, Perez-Harguindeguy N, Quested HM, Santiago LS, Wardle DA, Wright IJ, Aerts R, Allison SD, van Bodegom P, Brovkin V, Chatain A, Callaghan TV, Diaz S, Garnier E, Gurvich DE, Kazakou E, Klein JA, Read J, Reich PB, Soudzilovskaia NA, Vaieretti MV, Westoby M (2008). Plant species traits are the predominant control on litter decomposition rates within biomes worldwide. Ecol Lett.

[5] Djukic I, Kepfer-Rojas S, Schmidt IK, Larsen KS, Beier C, Berg B, Verheyen K, TeaComposition,  (2018). Early stage litter decomposition across biomes. Sci Total Environ.

[6] Krishna MP, Mohan M (2017). Litter decomposition in forest ecosystems: a review. Energy, Ecology and Environment.

[7] Zhang H, Thygesen LG, Mortensen K, Kádár Z, Lindedam J, Jørgensen H, Felby C (2014) Structure and enzymatic accessibility of leaf and stem from wheat straw before and after hydrothermal pretreatment. Biotechnol Biofuels 7. 10.1186/1754-6834-7-74 10.1186/1754-6834-7-74 PMC4031911 24860617

[8] Freschet GT, Cornwell WK, Wardle DA, Elumeeva TG, Liu W, Jackson BG, Onipchenko VG, Soudzilovskaia NA, Tao J, Cornelissen JHC, Austin A (2013). Linking litter decomposition of above- and below-ground organs to plant-soil feedbacks worldwide. J Ecol.

[9] Purahong W, Krüger D, Buscot F, Wubet T (2016). Correlations between the composition of modular fungal communities and litter decomposition-associated ecosystem functions. Fungal Ecol.

[10] Bani A, Pioli S, Ventura M, Panzacchi P, Borruso L, Tognetti R, Tonon G, Brusetti L (2018). The role of microbial community in the decomposition of leaf litter and deadwood. Appl Soil Ecol.

[11] Glassman SI, Weihe C, Li J, Albright MBN, Looby CI, Martiny AC, Treseder KK, Allison SD, Martiny JBH (2018). Decomposition responses to climate depend on microbial community composition. Proc Natl Acad Sci U S A.

[12] Zhao S, Zhang S (2018). Linkages between straw decomposition rate and the change in microbial fractions and extracellular enzyme activities in soils under different long-term fertilization treatments. PLoS ONE.

[13] Romaní AM, Fischer H, Mille-Lindblom C, Tranvik LJ (2006). Interactions of bacteria and fungi on decomposing litter: differential extracellular enzyme activities. Ecology.

[14] Hiscox J, Savoury M, Muller CT, Lindahl BD, Rogers HJ, Boddy L (2015). Priority effects during fungal community establishment in beech wood. ISME J.

[15] Fukami T, Dickie IA, Paula Wilkie J, Paulus BC, Park D, Roberts A, Buchanan PK, Allen RB (2010). Assembly history dictates ecosystem functioning: evidence from wood decomposer communities. Ecol Lett.

[16] Bradford MA, Warren Ii RJ, Baldrian P, Crowther TW, Maynard DS, Oldfield EE, Wieder WR, Wood SA, King JR (2014). Climate fails to predict wood decomposition at regional scales. Nat Clim Chang.

[17] Garcia-Palacios P, Maestre FT, Kattge J, Wall DH (2013). Climate and litter quality differently modulate the effects of soil fauna on litter decomposition across biomes. Ecol Lett.

[18] Cai A, Liang G, Zhang X, Zhang W, Li L, Rui Y, Xu M, Luo Y (2018). Long-term straw decomposition in agro-ecosystems described by a unified three-exponentiation equation with thermal time. Sci Total Environ.

[19] Yin R, Eisenhauer N, Auge H, Purahong W, Schmidt A, Schädler M (2019). Additive effects of experimental climate change and land use on faunal contribution to litter decomposition. Soil Biol Biochem.

[20] Schädler M, Buscot F, Klotz S, Reitz T, Durka W, Bumberger J, Merbach I, Michalski SG, Kirsch K, Remmler P, Schulz E, Auge H (2019) Investigating the consequences of climate change under different land-use regimes: a novel experimental infrastructure. Ecosphere 10:e02635. 10.1002/ecs2.2635

[21] Wahdan S, Hossen S, Tanunchai B, Schädler M, Buscot F, Purahong W (2020) Future climate significantly alters fungal plant pathogen dynamics during the early phase of wheat litter decomposition. Microorganisms 8. 10.3390/microorganisms8060908 10.3390/microorganisms8060908 PMC7356542 32560135

[22] Velásquez AC, Castroverde CDM, He SY (2018). Plant-pathogen warfare under changing climate conditions. Curr Biol.

[23] Rockel B, Will A, Hense A (2008). The regional climate model COSMO-CLM (CCLM). Meteorol Z.

[24] Jacob D, Podzun R (1997). Sensitivity studies with the regional climate model REMO. Meteorol Atmos Phys.

[25] Döscher R, Willén U, Jones C, Rutgersson A, Meier HEM, Hansson U, Graham LP (2002). The development of the regional coupled ocean-atmosphere model RCAO. Boreal Environ Res.

[26] Beier C, Emmett B, Gundersen P, Tietema A, Peñuelas J, Estiarte M, Gordon C, Gorissen A, Llorens L, Roda F, Williams D (2004) Novel approaches to study climate change effects on terrestrial ecosystems in the field: drought and passive nighttime warming. Ecosystems 7. 10.1007/s10021-004-0178-8

[27] Chelius MK, Triplett EW (2001). The diversity of archaea and bacteria in association with the roots of *Zea** mays* L. Microb Ecol.

[28] Bodenhausen N, Horton MW, Bergelson J (2013). Bacterial communities associated with the leaves and the roots of *Arabidopsis thaliana*. PLoS ONE.

[29] Beckers B, Op De Beeck M, Thijs S, Truyens S, Weyens N, Boerjan W, Vangronsveld J (2016). Performance of 16s rDNA primer pairs in the study of rhizosphere and endosphere bacterial microbiomes in metabarcoding studies. Front Microbiol.

[30] White TJ, Bruns TD, Lee SB, Taylor JW, Innis MA, Gelfand DH, Sninsky JJ, White TJ (1990). Amplification and direct sequencing of fungal ribosomal RNA genes for phylogenetics. PCR protocols: a guide to methods and applications.

[31] Martin M (2011). Cutadapt removes adapter sequences from high-throughput sequencing reads. EMBnet J.

[32] Masella AP, Bartram AK, Truszkowski JM, Brown DG, Neufeld JD (2012) PANDAseq: paired-end assembler for illumina sequences. BMC Bioinformatics 13. 10.1186/1471-2105-13-31 10.1186/1471-2105-13-31 PMC3471323 22333067

[33] Edgar RC, Haas BJ, Clemente JC, Quince C, Knight R (2011). UCHIME improves sensitivity and speed of chimera detection. Bioinformatics.

[34] Fu L, Niu B, Zhu Z, Wu S, Li W (2012). CD-HIT: accelerated for clustering the next-generation sequencing data. Bioinformatics.

[35] Quast C, Pruesse E, Yilmaz P, Gerken J, Schweer T, Yarza P, Peplies J, Glockner FO (2013). The SILVA ribosomal RNA gene database project: improved data processing and web-based tools. Nucleic Acids Res.

[36] Kõljalg U, Nilsson RH, Abarenkov K, Tedersoo L, Taylor AFS, Bahram M, Bates ST, Bruns TD, Bengtsson-Palme J, Callaghan TM, Douglas B, Drenkhan T, Eberhardt U, Dueñas M, Grebenc T, Griffith GW, Hartmann M, PlM K, Kohout P, Larsson E, Lindahl BD, Lücking R, Martín MP, Matheny PB, Nguyen NH, Niskanen T, Oja J, Peay KG, Peintner U, Peterson M, Põldmaa K, Saag L, Saar I, Schüßler A, Scott JA, Senés C, Smith ME, Suija A, Taylor DL, Telleria MT, Weiß M, Larsson K-H (2013). Towards a unified paradigm for sequence-based identification of fungi. Mol Ecol.

[37] Schloss PD, Westcott SL, Ryabin T, Hall JR, Hartmann M, Hollister EB, Lesniewski RA, Oakley BB, Parks DH, Robinson CJ, Sahl JW, Stres B, Thallinger GG, Van Horn DJ, Weber CF (2009). Introducing mothur: open-source, platform-independent, community-supported software for describing and comparing microbial communities. Appl Environ Microbiol.

[38] Kunin V, Engelbrektson A, Ochman H, Hugenholtz P (2010). Wrinkles in the rare biosphere: pyrosequencing errors can lead to artificial inflation of diversity estimates. Environ Microbiol.

[39] Louca S, Parfrey LW, Doebeli M (2016). Decoupling function and taxonomy in the global ocean microbiome. Science.

[40] Nguyen NH, Song Z, Bates ST, Branco S, Tedersoo L, Menke J, Schilling JS, Kennedy PG (2016). FUNGuild: an open annotation tool for parsing fungal community datasets by ecological guild. Fungal Ecol.

[41] Gutiérrez Boem FH, Rubio G, Barbero D (2011). Soil phosphorus extracted by Bray 1 and Mehlich 3 soil tests as affected by the soil/solution ratio in Mollisols. Commun in Soil Sci Plant Anal.

[42] Purahong W, Durka W, Fischer M, Dommert S, Schops R, Buscot F, Wubet T (2016). Tree species, tree genotypes and tree genotypic diversity levels affect microbe-mediated soil ecosystem functions in a subtropical forest. Sci Rep.

[43] Hammer Ø, Harper DAT, Ryan PD (2001) PAST: Paleontological statistics software package for education and data analysis. Palaeontol Elec 4:1–9

[44] Anderson MJ (2001). A new method for non-parametric multivariate analysis of variance. Aus Ecol.

[45] Jarque CM, Lovric M (2011). Jarque-Bera Test. International encyclopedia of statistical science.

[46] Shannon P, Markiel A, Ozier O, Baliga NS, Wang JT, Ramage D, Amin N, Schwikowski B, Ideker T (2003). Cytoscape: a software environment for integrated models of biomolecular interaction networks. Genome Res.

[47] Steele JA, Countway PD, Xia L, Vigil PD, Beman JM, Kim DY, Chow CE, Sachdeva R, Jones AC, Schwalbach MS, Rose JM, Hewson I, Patel A, Sun F, Caron DA, Fuhrman JA (2011). Marine bacterial, archaeal and protistan association networks reveal ecological linkages. ISME J.

[48] Agler MT, Ruhe J, Kroll S, Morhenn C, Kim ST, Weigel D, Kemen EM (2016). Microbial hub taxa link host and abiotic factors to plant microbiome variation. PLoS Biol.

[49] Classen AT, Sundqvist MK, Henning JA, Newman GS, Moore JAM, Cregger MA, Moorhead LC, Patterson CM (2015) Direct and indirect effects of climate change on soil microbial and soil microbial-plant interactions: What lies ahead? Ecosphere 6. 10.1890/ES15-00217.1

[50] Bradford MA (2013). Thermal adaptation of decomposer communities in warming soils. Front Microbiol.

[51] Schimel J, Balser TC, Wallenstein M (2007). 10.1890/06-0219. Ecology.

[52] Vetrovsky T, Kohout P, Kopecky M, Machac A, Man M, Bahnmann BD, Brabcova V, Choi J, Meszarosova L, Human ZR, Lepinay C, Llado S, Lopez-Mondejar R, Martinovic T, Masinova T, Morais D, Navratilova D, Odriozola I, Stursova M, Svec K, Tlaskal V, Urbanova M, Wan J, Zifcakova L, Howe A, Ladau J, Peay KG, Storch D, Wild J, Baldrian P (2019). A meta-analysis of global fungal distribution reveals climate-driven patterns. Nat Commun.

[53] Martiny JB, Martiny AC, Weihe C, Lu Y, Berlemont R, Brodie EL, Goulden ML, Treseder KK, Allison SD (2017). Microbial legacies alter decomposition in response to simulated global change. ISME J.

[54] Rowley MC, Grand S, Verrecchia ÉP (2017). Calcium-mediated stabilisation of soil organic carbon. Biogeochemistry.

[55] Kerdraon L, Laval V, Suffert F (2019). Microbiomes and pathogen survival in crop residues, an ecotone between plant and soil. Phytobiomes Journal.

[56] Rezig FAM, Elhadi EA, Abdalla MR (2014) Decomposition and nutrient release pattern of wheat (*Triticum aestivum*) residues under different treatments in desert field conditions of Sudan. Int J Recycling Organic Waste Agric 3. 10.1007/s40093-014-0069-8

[57] Rubenstein MA, Crowther TW, Maynard DS, Schilling JS, Bradford MA (2017). Decoupling direct and indirect effects of temperature on decomposition. Soil Biol Biochem.

[58] Kerdraon L, Barret M, Laval V, Suffert F (2019) Differential dynamics of microbial community networks help identify microorganisms interacting with residue-borne pathogens: the case of *Zymoseptoria tritici* in wheat. Microbiome 7:125. 10.1186/s40168-019-0736-0 10.1186/s40168-019-0736-0 PMC6717385 31470910

[59] Pessoa MG, Paulino BN, Mano MCR, Neri-Numa IA, Molina G, Pastore GM (2017). *Fusarium* species-a promising tool box for industrial biotechnology. Appl Microbiol Biotechnol.

