# Supplementary material for: Life in the Wheat Litter: Effects of Future Climate on Microbiome and Function During the Early Phase of Decomposition
Source: Microb Ecol. 2021 Sep 6;84(1):90–105. doi: 10.1007/s00248-021-01840-6 (PMC9250916; doi:10.1007/s00248-021-01840-6)
Supplement: Supplementary file 1 — Supplementary file1 (DOCX 4456 KB) [file 248_2021_1840_MOESM1_ESM.docx]

**Life in the wheat litter: effects of future climate on microbiome and function during the early phase of decomposition**

Sara Fareed Mohamed Wahdan ^1, 2, 3, ‡, *^, Shakhawat Hossen ^1, 4, 5 ‡^, Benjawan Tanunchai ^1^, Chakriya Sansupa ^1^, Martin Schädler ^6, 7^, Matthias Noll ^5^, Turki M. Dawoud ^3^, Yu-Ting Wu ^8, *^, François Buscot ^1, 3, 7, †^ and Witoon Purahong ^1, 3, †^

[sara-fareed-mohamed.wahdan@ufz.de](mailto:sara-fareed-mohamed.wahdan@ufz.de), sarah_wahdan@science.suez.edu.eg (Sara F. M. Wahdan); [shakhawat.hossen@ufz.de (Shakhawat Hossen)](mailto:shakhawat.hossen@ufz.de%20(Shakhawat%20Hossen)); [tanunchai.benjawan@ufz.de](mailto:tanunchai.benjawan@ufz.de) (Benjawan Tanunchai); [chakriya.sansupa@gmail.com](mailto:chakriya.sansupa@gmail.com) (Chakriya Sansupa); [martin.schaedler@ufz.de](mailto:martin.schaedler@ufz.de) (Martin Schädler); tdawoud@ksu.edu.sa (Turki M. Dawoud); yutingwu@mail.npust.edu.tw (Yuting Wu); [francois.buscot@ufz.de](mailto:francois.buscot@ufz.de) (François Buscot); [witoon.purahong@ufz.de](mailto:witoon.purahong@ufz.de) (Witoon Purahong).

**Affiliations:**

^1^ UFZ-Helmholtz Centre for Environmental Research, Department of Soil Ecology, Theodor-Lieser-Str. 4, 06120 Halle (Saale), Germany

^2^ Botany Department, Faculty of Science, Suez Canal University, 41522 Ismailia, Egypt

^3^ Botany and Microbiology Department, College of Science, King Saud University, P. O. Box 2455, 11451 Riyadh, Saudi Arabia

^4^ Friedrich-Schiller-Universität Jena, Institute of Ecology and Evolution, Dornburger Str. 159, 07743 Jena, Germany

^5^ Institute for Bioanalysis, Department of Applied Sciences, Coburg University of Applied Sciences and Arts, Coburg, Germany

^6^ UFZ-Helmholtz Centre for Environmental Research, Department of Community Ecology, Theodor-Lieser-Str. 4, 06120 Halle (Saale), Germany

^7^ German Centre for Integrative Biodiversity Research (iDiv) Halle-Jena-Leipzig, Deutscher Platz 5e, 04103 Leipzig, Germany

^8^ Department of Forestry, National Pingtung University of Science and Technology, Pingtung 91201, Taiwan

**^*^Correspondence:**

Sara F. M. Wahdan

[sara-fareed-mohamed.wahdan@ufz.de](mailto:sara-fareed-mohamed.wahdan@ufz.de)

Yu-Ting Wu

[yutingwu@mail.npust.edu.tw](mailto:yutingwu@mail.npust.edu.tw)

^†^Senior Authors.

^‡^These authors contributed equally to this work.

**Supplementary Tables**

**Table S1.** Physicochemical properties of GCEF plots soil of conventional farming ecosystem under ambient and future climate conditions at the onset of the experiment. Values represent mean ± sd. The values did not differ significantly between ambient and future climate soils (t-test, P > 0.05).

| **Edaphic/climatic factor** | **Current climate** | **Future climate** |
| --- | --- | --- |
| TOC (%) | 2.009±0.076 | 2.391±0.724 |
| TON (%) | 0.005±0.004 | 0.006±0.009 |
| C/N | 11.958±0.739 | 12.307±0.772 |
| pH | 6.796±0.475 | 6.852±0.617 |

**Table S2.** Results of the permutational multivariate analysis of variance (NPMANOVA) test analyzing the effects of decomposition time (sampling date), climate conditions, and the interaction between the two factors on the bacterial and fungal communities’ composition in wheat litter. Significant results (p < 0.05) from NPMANOVA are indicated in bold.

| **Tested factor** | **Bacterial community** | | **Fungal community** | |
| --- | --- | --- | --- | --- |
|  | *F*-value | *p*-value | *F*-value | *p*-value |
| Climate | 1.1318 | 0.148 | 1.4566 | **0.015** |
| Decomposition time | 2.2504 | **0.001** | 1.7648 | **0.001** |
| Interaction between climate and decomposition | 1.11 | 0.129 | 1.333 | **0.011** |
| **Ambient climate** | | | | |
| 0 days Χ 30 days | 1.515 | **0.01** | 1.264 | 0.097 |
| 30 days Χ 60 days | 1.066 | 0.171 | 0.8089 | 0.885 |
| 0 days Χ 60 days | 2.092 | **0.011** | 1.1 | 0.316 |
| **Future climate** | | | | |
| 0 days Χ 30 days | 1.517 | **0.007** | 1.305 | 0.05 |
| 30 days Χ 60 days | 1.609 | **0.009** | 2.176 | **0.008** |
| 0 days Χ 60 days | 2.33 | **0.008** | 2.811 | **0.01** |

**Table S3.** Co-presence and exclusion correlations of fungal pathogens and other microbes under ambient and future climate conditions, see file: supplementary_file_S3&S4.xlsx

**Table S4.** Classification of bacterial and fungal operational taxonomic units (OTUs) correlated with fungal pathogens in ambient and future co-occurrence networks, see file: supplementary_file_S3&S4.xlsx

**Supplementary Figures**


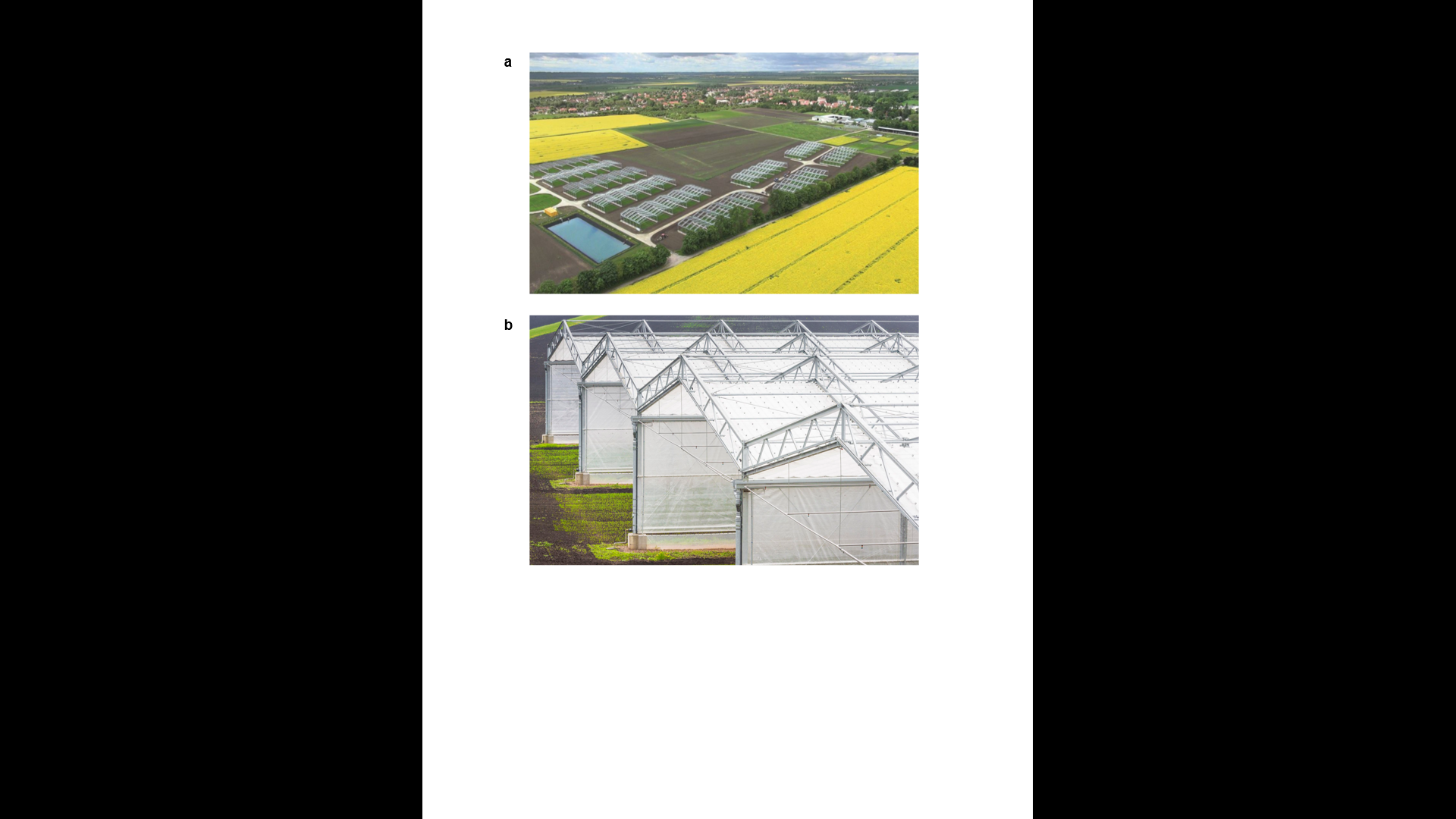


**Figure S1.** (**a**) Aerial view for the Global Change Experimental Facility (GCEF) field research station of the Helmholtz Centre for Environmental Research in Bad Lauchstädt, Saxony-Anhalt, Germany, photo taken by Tricklabor Berlin/Service Drone. (**b**) Closed shelters and panels of the future climate plots of the GCEF, photo taken by UFZ/ André Künzelmann.

**Figure S2.** Effects of climate manipulation on (A) total precipitation (sum of season) and (B) soil temperature (daily mean temperature) in a depth of 1 cm in experimental plots managed by conventional farming in the GCEF. Precipitation is not manipulated during the winter months. Note that the effects of soil temperature is strongly modulated by indirect effects via the change of vegetation cover (see also Schädler *et al.* 2019). Here, better growing conditions during the establishment of winter rape (autumn-spring) in the future treatment (higher precipitation, warmer) led to a higher vegetation cover and consequently lower direct insolation and lower soil temperatures in this treatment. This could not be observed for winter wheat in 2018 since plant growth was generally weak due to the generally low amount of precipitation.


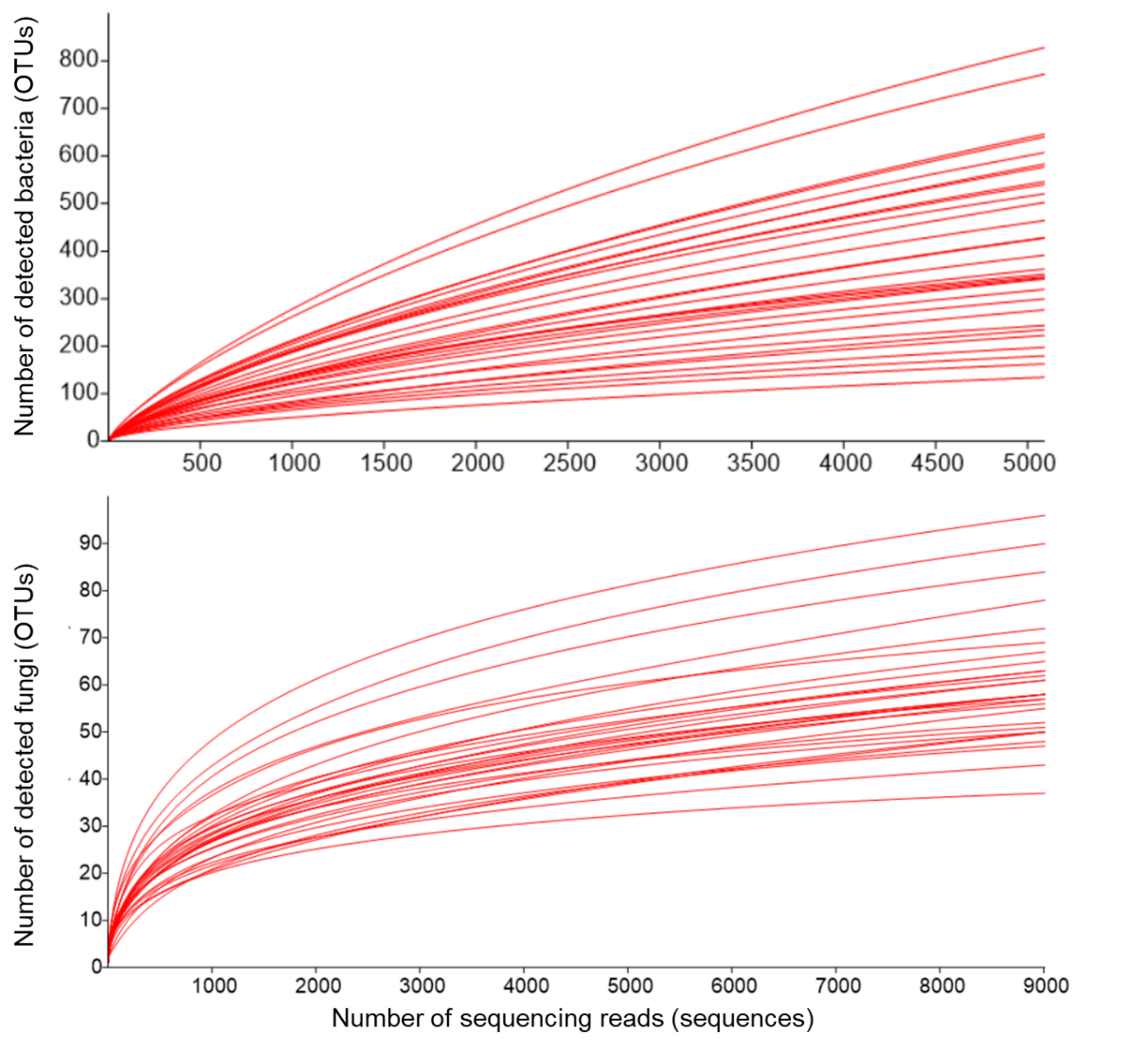


**Figure S3.** Samples rarefaction curves of the sequencing reads for bacteria and fungi detected in sampling times at the early phase of wheat litter decomposition.


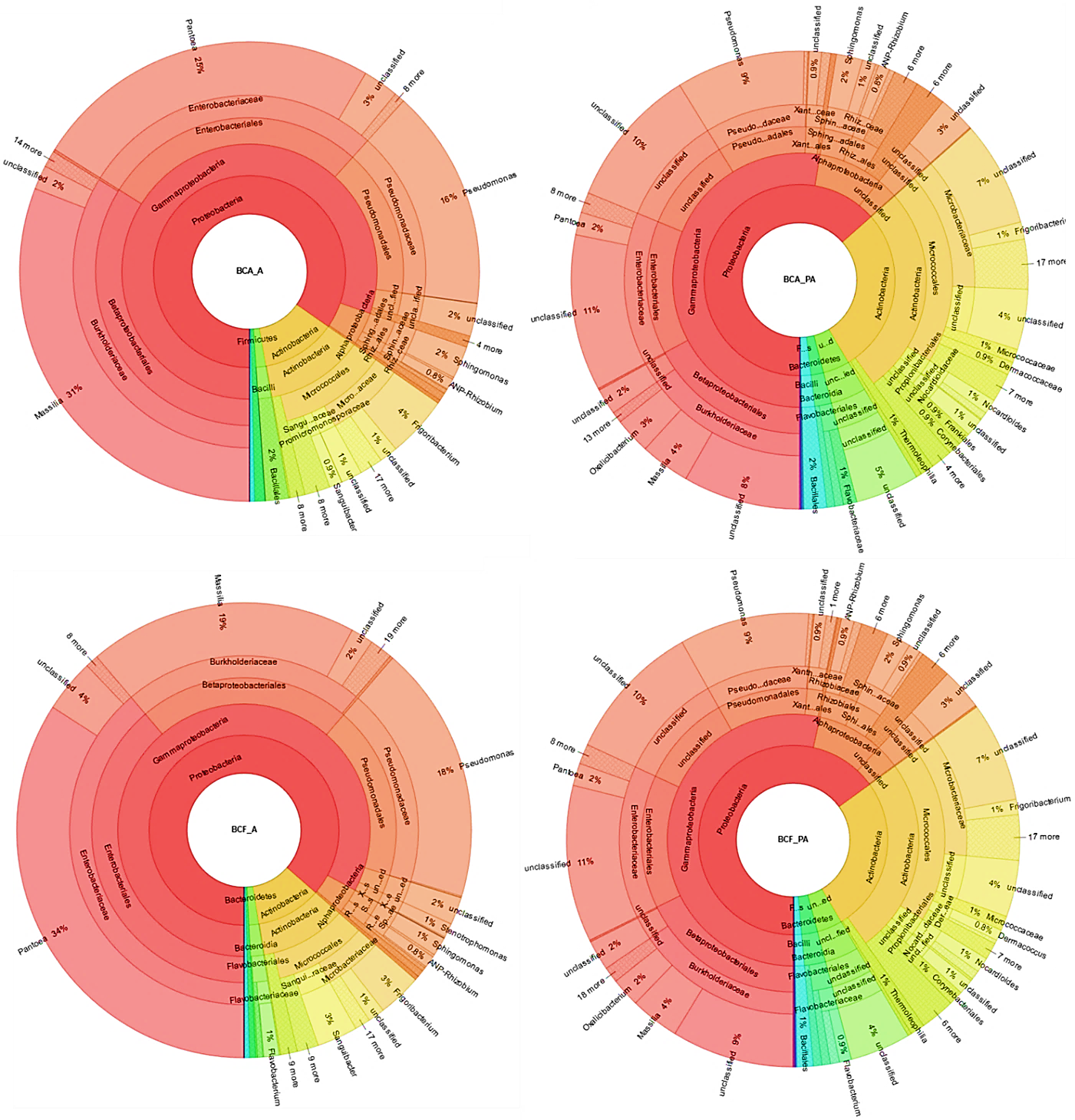


**Figure S4.** Bacterial community compositions associated with wheat litter samples at the early phase of decomposition in ambient and future climate treatments based on relative abundances and presence/absence data. BCA_A = bacterial community composition in ambient climate based on relative abundances data, BCA_PA = bacterial community composition in ambient climate based on presence/absence data.


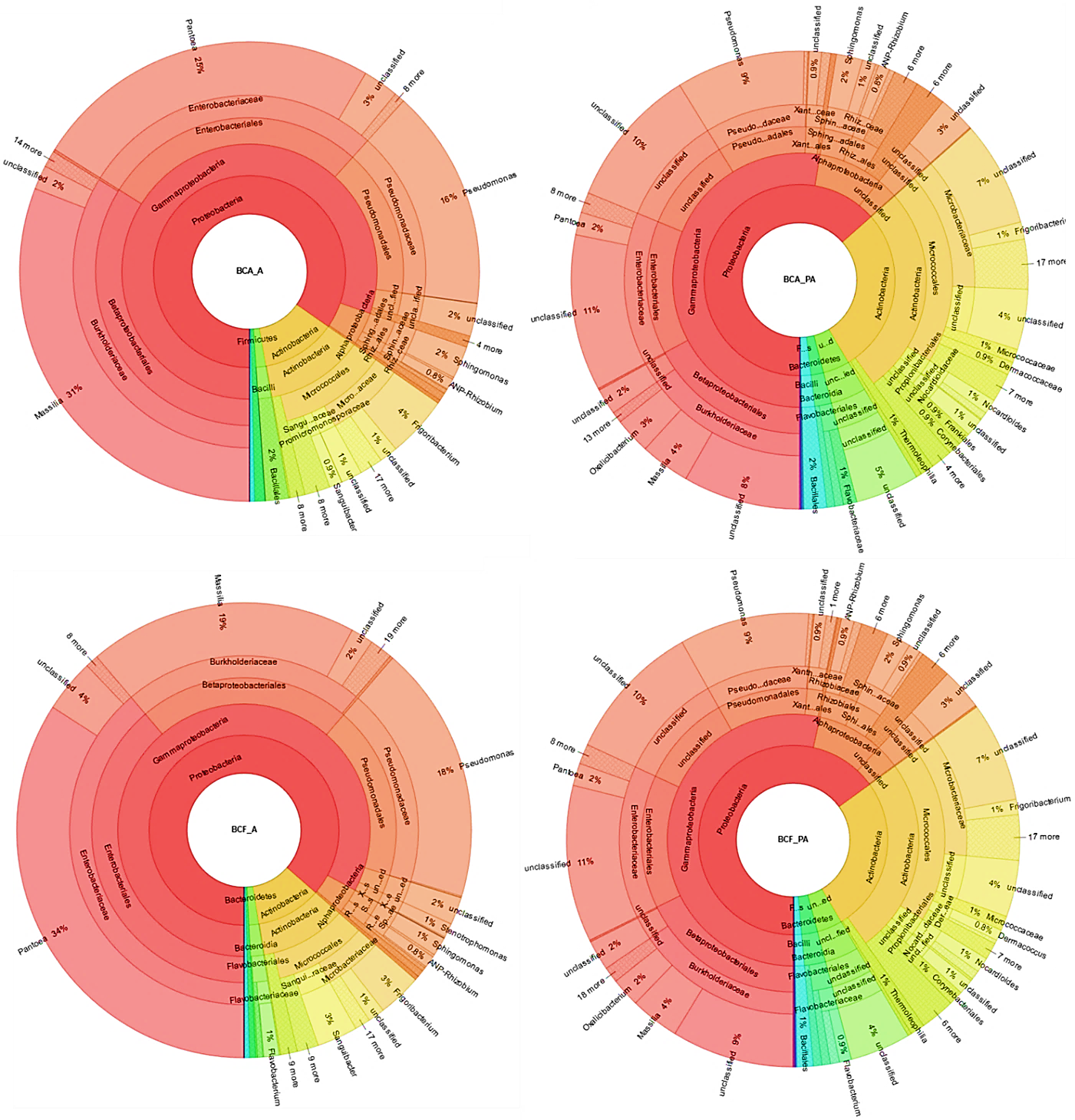


**Figure S4.** continued

**
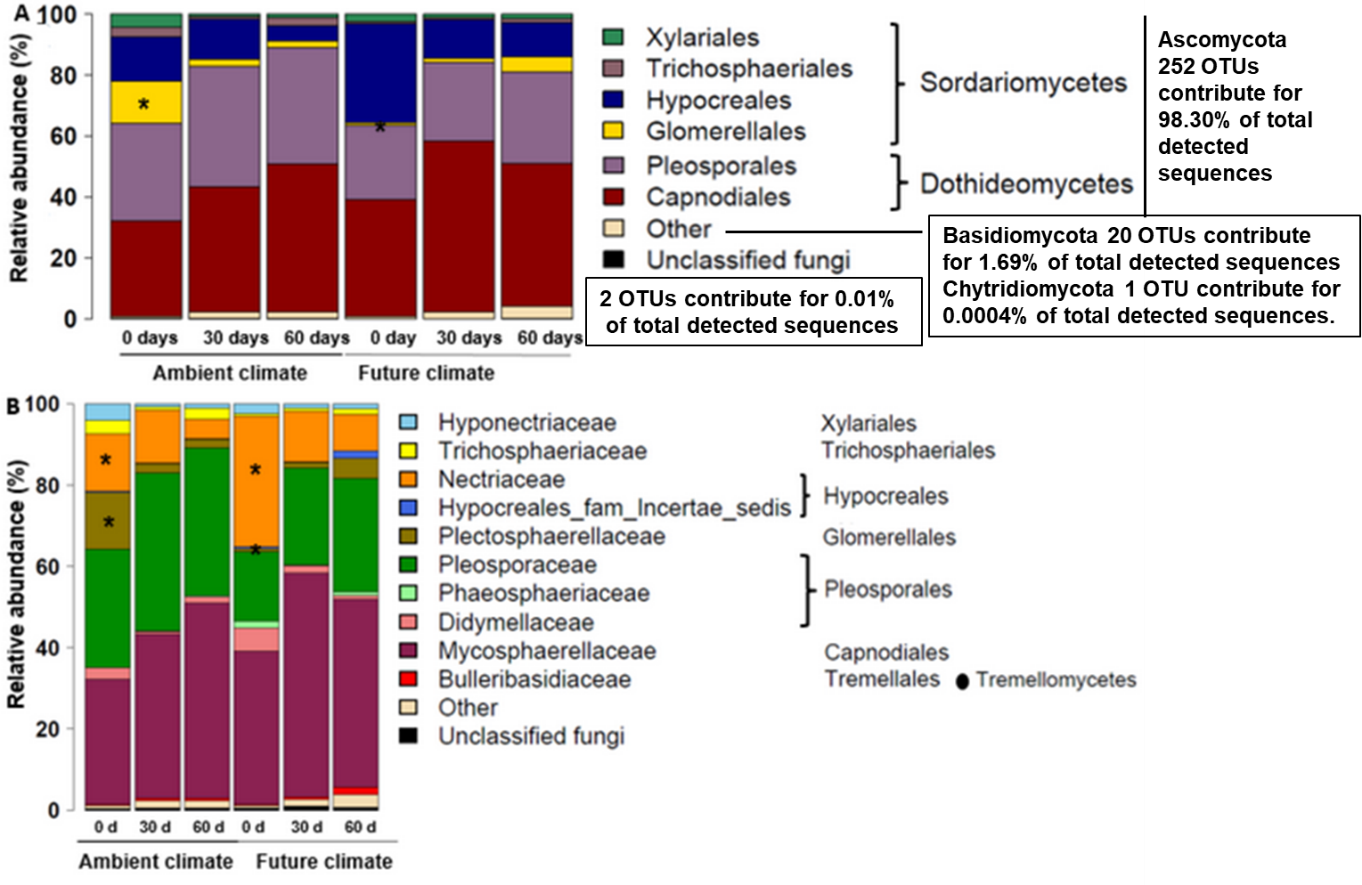
**

**Figure S5.** Fungal community compositions associated with wheat litter samples at the early phase of decomposition in ambient and future climate treatments (reproduced from Wahdan *et al.*, (2020)).


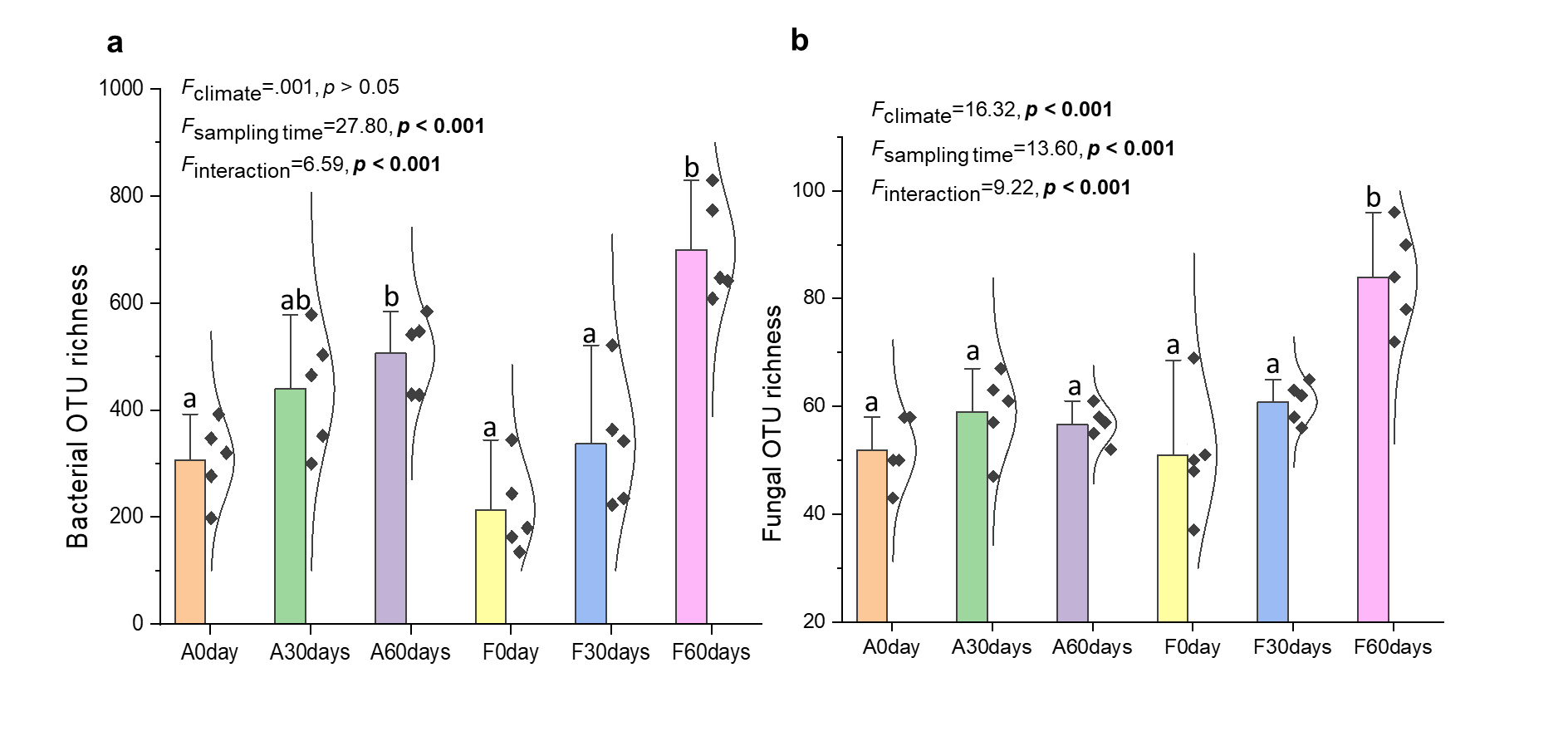


**Figure S6.** Effect of climate, time and their interaction on bacterial (**a**) and fungal (**b**) richness at the early stage of wheat litter decomposition. Significant results following Tukeys HSD at *p* < 0.05 are indicated in bold. In the bar plots, the letters A and F represent ambient and future climate treatments, respectively.

**
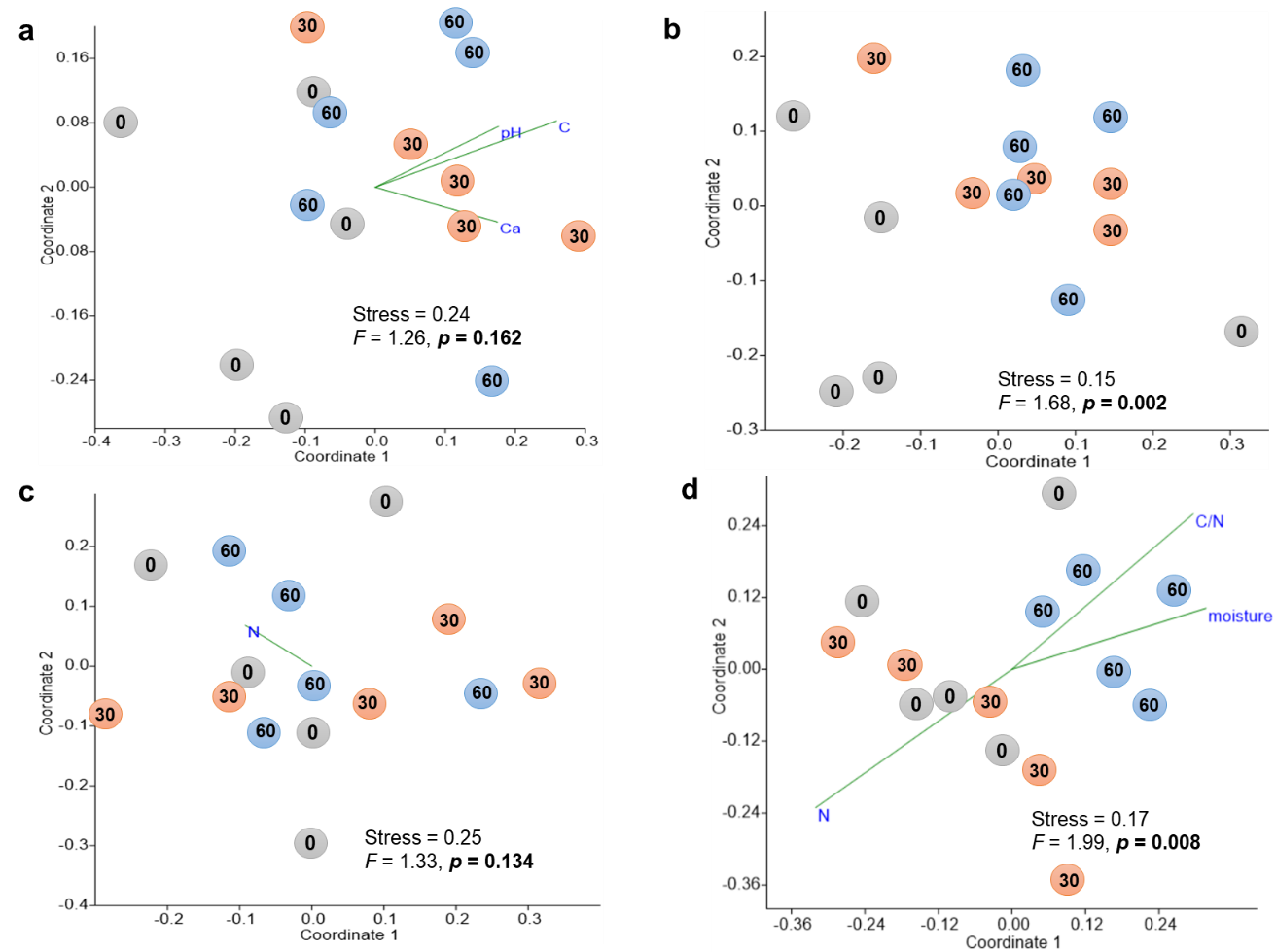
**

**Figure S7.** Non-metric multidimensional scaling (NMDS) ordination diagrams of the potential plant pathogenic (**ambient: a; future: b**) and saprotrophic (**ambient: c future: d**) fungal communities colonizing wheat litter residues under ambient and future climate condition. NMDS ordination based on Jaccard dissimilarities was used to determine the compositional variation over sampling time. In NMDS ordinations, the numbers 0, 30 and 60 represent the sampling time in days. All significant wheat litter physicochemical properties (*p* < 0.05) were plotted in the respective NMDS ordination plots. Significant effect of time (*p* < 0.05) based on NPMANOVA are indicated in bold.
